# Mechanistic Insights into CO_2_‑to-CO Photoreduction by Proton-Responsive Imidazole–Pyridine Re(I) Complexes

**DOI:** 10.1021/acs.inorgchem.6c00967

**Published:** 2026-04-16

**Authors:** Marcos E. G. Carmo, Gabrielly Moreira, Vanessa F. Silva, Jaqueline C. Desordi, Pablo J. Gonçalves, Antonio E. Hora Machado, Renato N. Sampaio, Gerald J. Meyer, Antonio O. T. Patrocinio

**Affiliations:** † Laboratory of Photochemistry and Materials Science, Institute of Chemistry, Federal University of Uberlândia, Uberlândia, Minas Gerais 38400-902,Brazil; ‡ Centro de Excelência em Hidrogênio e Tecnologias Energéticas Sustentáveis (CEHTES), Goiânia, Goiás 74690-631, Brazil; § Instituto de Física, Universidade Federal de Goiás, Goiânia 74690-900, Brazil; ∥ Programa de Doutorado em Ciências Exatas e Tecnológicas, Universidade Federal de Catalão − UFCat, Catalão, Goiás 75704-020, Brazil; ⊥ Department of Chemistry, 2331 University of North Carolina at Chapel Hill, Chapel Hill, North Carolina 27599, United States

## Abstract

Two Re­(I) tricarbonyl complexes with imidazole-pyridine ligands, *fac*-[Re­(CO)_3_(pbiH)­Cl], (Re-pbiH), and *fac*-[Re­(CO)_3_(bbzp)­Cl] (Re-bbzp), where pbiH= 2-(2-pyridy)­benzimidazole, bbzp = 2,6-bis­(2-benzimidazolyl)­pyridine, were synthesized, and their photophysical, electrochemical, and photochemical properties were investigated for application as photocatalysts for CO_2_-to-CO reduction. Upon light irradiation (λ > 370 nm) in CO_2_-saturated CH_3_CN using 1,3-dimethyl-2-phenyl-2,3-dihydro-1*H*-benzo­[d]­imidazole (BIH) as a sacrificial donor, Re-bbzp promotes CO formation with a turnover number (TON_CO_) of 45 ± 2, whereas Re-pbiH displayed a significantly lower activity (TON_CO_ = 8 ± 1). The role of the bbzp ligand in the photocatalytic behavior was examined in detail using IR spectroelectrochemistry (IR-SEC) together with *in situ* FTIR and UV–vis spectroscopy under photocatalytic conditions, revealing the formation of key intermediates involved in CO_2_ activation. The superior activity of Re-bbzp was rationalized by its ability to function as a proton relay and two-electron acceptor. The role as a proton relay was further supported by the observation of a kinetic isotope effect (KIE = 1.5) when the deuterated electron donor BID was employed, in agreement with the unusual decrease in catalytic activity observed upon addition of Brønsted bases. Overall, these results provide new insights into the role of ligand-based proton-responsive sites in Re­(I) tricarbonyl complexes and their impact on CO_2_ reduction photocatalysis.

## Graphical abstract

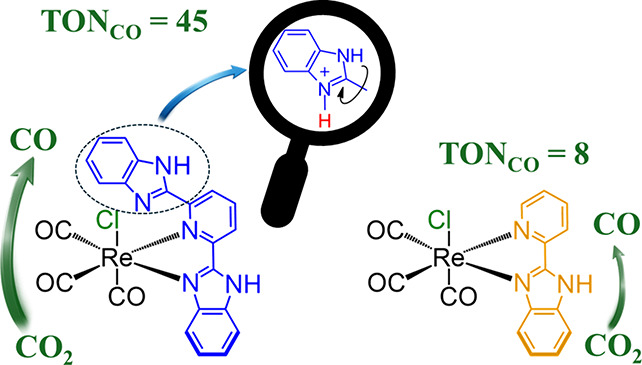

## Introduction

Re­(I) tricarbonyl complexes have been extensively investigated as electro/photocatalysts for CO_2_ reduction to CO. Since the first report by Lehn et al. employing the parental complex *fac-*[Re­(CO)_3_(bpy)­Cl], bpy = 2,2′-bipyridine, subsequent studies have investigated the reaction mechanisms and the role of the polypyridyl ligand on the catalytic processes.^1–10^ The irradiation of such complexes typically gives rise to metal-to-ligand charge transfer (MLCT) excited states characterized by relatively long lifetimes and suitable energetics for CO_2_-to-CO conversion.^11,12^

In photoinduced CO_2_ reduction, a sacrificial donor is usually necessary. Triethanolamine (TEOA) has typically been employed, but Ishitani et al. have shown that the use of benzomidazole derivatives, such as 1,3-dimethyl-2-phenyl-2,3-dihydro*-*1*H-*benzo­[d]­imidazole (BIH), can lead to better photocatalytic performance. As shown in Scheme S1, BIH is capable of donating two electrons and one proton, which also has a stronger reducing power compared to triethanolamine (TEOA) and triethylamine (TEA) as discussed in previous works.^100–16^ Additionally, the presence of a Brønsted acid in the reaction medium or in the catalysts’s secondary coordination sphere is also beneficial for photocatalysis.^17^ In the accepted catalytic cycle, CO_2_ coordination to the reduced catalyst is followed by proton transfer to yield a Re-CO_2_H intermediate that is further reduced and protonated to yield CO and H_2_O.^18,19^ The presence of a proton relay close to the metal center may stabilize an intermediate and improve reaction rates as observed for other homogeneous catalysts.^20–24^

Re­(I)-based imidazole-pyridyl complexes combine excited-state redox chemistry with a proton relay close to the metal center. Such intriguing properties have been previously exploited. For example, Qiu et al. reported a series of Re­(I) complexes with an imidazole-pyridine framework in which a π-extended pyrene was covalently bonded to the N atom of the imidazole ring. The complexes exhibited extended triplet excited-state lifetimes and high photoactivity for CO_2_ reduction.^11^ Siewert and co-workers reported a series of binuclear Re­(I) complexes with imidazole-pyridyl ligands^25,26^ as well as pyrazole-pyridyl ligands^27^ in which the role of the proton-responsive groups was explored for electrochemical CO_2_ reduction. Furthermore, Warren and co-workers reported imidazole-based Re­(I) complexes in which alkalization of the imidazole proton resulted in loss of catalytic activity toward CO_2_ reduction, evidencing the role of ligand-centered proton acceptor sites on CO_2_ activation.^28^ Moreover, Sinha et al. reported CO_2_ reduction by *fac-*[Re­(CO)_3_(bbzp)­Cl], bbzp = 2,6-bis­(2-benzimidazolyl)­pyridine in aqueous media.^29,30^ The role of the pendant nonchelating benzimidazolyl group was compared with a Re­(I) complex bearing a substituted terpyridine ligand. All of these reports emphasize the importance of the imidazole group(s) in enhancing stability and reactivity; however, to our knowledge, prior work has not explored the photophysical and photochemical properties of the catalysts or their catalytic mechanism during the CO_2_ reduction cycle.

In this work, the photophysical properties of two Re­(I) tricarbonyl complexes, *fac-*[Re­(CO)_3_(pbiH)­Cl] (Re-pbiH) and *fac-*[Re­(CO)_3_(bbzp)­Cl] (Re-bbzp), pbiH = 2,6-bis­(2-benzimidazolyl)­pyridine, Figure 1, are reported. The role of the additional benzimidazolyl group of the bbzp ligand is described along with their performance as a photocatalyst in the CO_2_ reduction reaction (CO_2_RR). The results bring new insights into the role of the pendant N-imidazolyl atom as a proton relay for the reduction of CO_2_ to CO.

**Figure 1:**
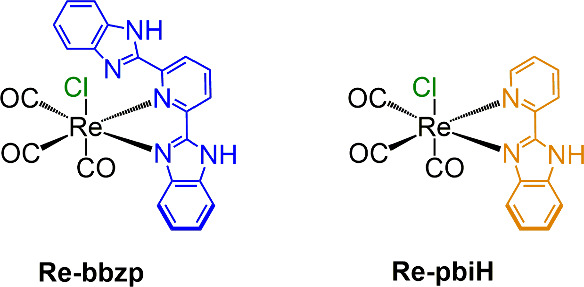
Chemical structures and abbreviations of the Re­(I) catalysts.

## Experimental Methods

The complexes were prepared by refluxing [ClRe­(CO)_5_] and the respective ligands in toluene. Detailed description of the synthetic procedures is given in the Supporting Information along with characterization data and other experimental and computational details.

### Photocatalytic Studies

CO_2_ photoreduction experiments were carried out in a closed double-jacket glass reactor with a total volume of 16 mL. The reactor was loaded with 10 mL of a 0.03 mmol L^–1^ acetonitrile solution of the catalyst and an excess of 6.7 mmol L^–1^ mg of BIH. The mixture was then purged with CO_2_ until saturation and exposed to a 300 W Xe lamp equipped with a water filter and a 370 nm long-pass filter with an irradiance of 100 mW cm^–2^. The reactor was kept at 25 °C with a thermally controlled water circulator. At given time intervals, 100 μL of the headspace atmosphere was sampled using a gastight syringe and analyzed by gas chromatography (Shimadzu GC2014 equipped with a flame ionization detector (FID) and a methanizer) to quantify CO formation. A 500 μL aliquot was analyzed for H_2_ with a PerkinElmer Clarus 580–GC instrument equipped with a thermal conductivity detector. Photocatalytic experiments were also performed in a custom reactor illuminated with a Newport Oriel 1 kW Solar Simulator (model 91191-1000; 900 W Xe lamp and LP370 long-pass filter) connected in-line to the sample loop of a gas chromatograph (Agilent GC 8890). Gaseous products were separated with a carbonPLOT column and a molecular sieve column in series. The carbonPLOT column decelerated the elution of CO_2_ relative to the other lighter gases (O_2_, N_2_, H_2_, and CO), while the molecular sieves separated each gas with specific retention times. A thermal conductivity detector (TCD) was used to detect O_2_, N_2_, and H_2_. A methanizer equipped with a nickel hydrogenation catalyst was paired with a flame ionization detector (FID) to detect CO_2_ and CO. Argon (Ar) was used as the carrier gas.

### FTIR Spectroelectrochemical (IR-SEC) Experiments

IR spectroelectrochemical studies were performed using an optically transparent thin-layer electrochemical (OTTLE) cell with a calcium fluoride (CaF_2_) window. All spectroelectrochemical experiments were conducted in 0.1 M TBAH/acetonitrile solutions under an Ar/CO_2_ atmosphere. Blank acetonitrile solutions with 0.1 M TBAH were used as the background. A PerkinElmer Frontier spectrometer was used to monitor thin-layer bulk electrolysis coupled to an μAutolab PGSTAT204 potentiostat/galvanostat (Autolab). A platinum grid working electrode, platinum wire counter electrode, and silver wire pseudoreference electrode were employed for the chronoamperometric studies.

### *In Situ* FTIR Experiments

The *in situ* FTIR experiment was performed by using an optically transparent thin-layer electrochemical (OTTLE) cell with a sodium chloride (NaCl) window. All *in situ* experiments were performed in solutions containing 0.03 mmol L^–1^ of photocatalyst and an excess of 6.7 mmol L^–1^ of BIH in acetonitrile under Ar/CO_2_ atmosphere. The PerkinElmer Frontier spectrometer was employed to monitor the formed intermediates as a function of time (λ_irr_ >370 nm; *P*_irr_ = 100 mW cm^–2^).

## Results and Discussion

### Structural Characterization

The synthesis of both complexes was successfully achieved as confirmed by ^1^H NMR (Figure 2), elementary analysis, and mass spectrometry (Figures S1 and S2).^29,30^ The ^1^H NMR spectra of both complexes are characterized by peaks in the aromatic region, which are downfield from those observed for the free ligands due to coordination to the Re­(I) center. The peak positions and their corresponding coupling constants agree well with those previously reported for these complexes.^29,30^ Additionally, between 13 and 15 ppm, broad singlet peaks observed for both species are attributed to the hydrogen atom bonded to the N atom in the imidazole ring coordinated to the Re­(I) center. For the Re-bbzp complex, one additional singlet is observed at 13.3 ppm, corresponding to the pendant imidazole ring. The broad shape of these peaks provides evidence of their acidic character.^31^ As expected, the interaction of the imidazole ring with the Re­(I) center leads to an increase in the Brønsted acid character of the neighboring NH group, which becomes more downshielded.

**Figure 2:**
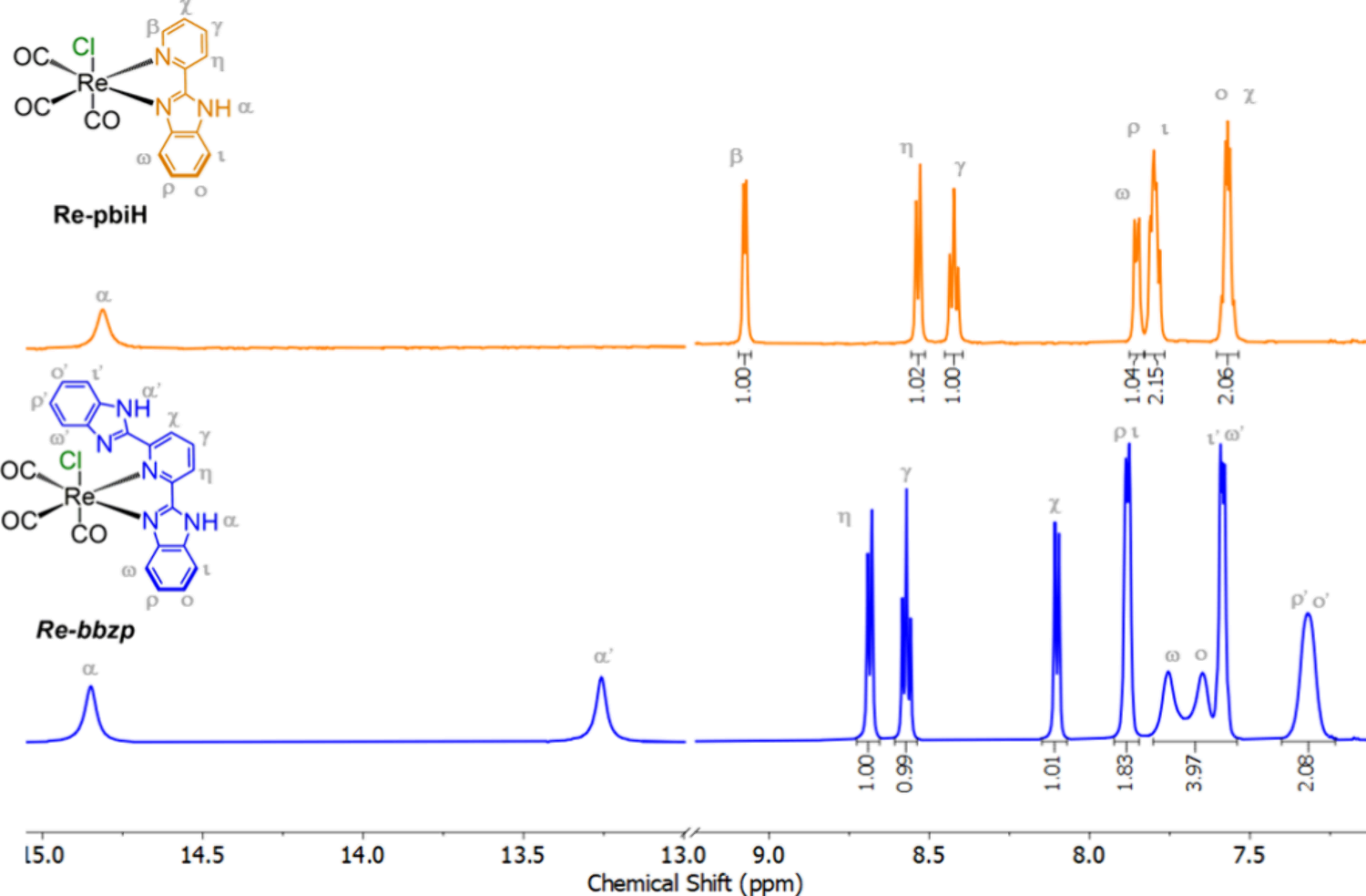
^1^H NMR spectra of Re-pbiH (orange) and Re-bbzp (blue) in DMSO-d^6^.

### Photophysical Properties

The electronic absorption spectra of both complexes were measured in CH_3_CN at 298 K (Figure 3a). For both complexes, highly intense absorption bands (ε >10^4^ L mol^–1^ cm^–1^) between 240 and 360 nm are observed and attributed to allowed intraligand (IL_π→π*_) transitions. At lower energies (λ >360 nm), an absorption tail is observed, corresponding to a metal-to-ligand charge transfer (MLCT) transition.^32–35^ For the Re–pbiH complex, the MLCT band is clearly observed with a maximum at 375 nm (ε = 4.2 × 10^3^ L mol^–1^ cm^–1^). In contrast, for Re-bbzp, the MLCT and IL bands overlap in energy due to the extended π-conjugation of the bbzp ligand.

**Figure 3:**
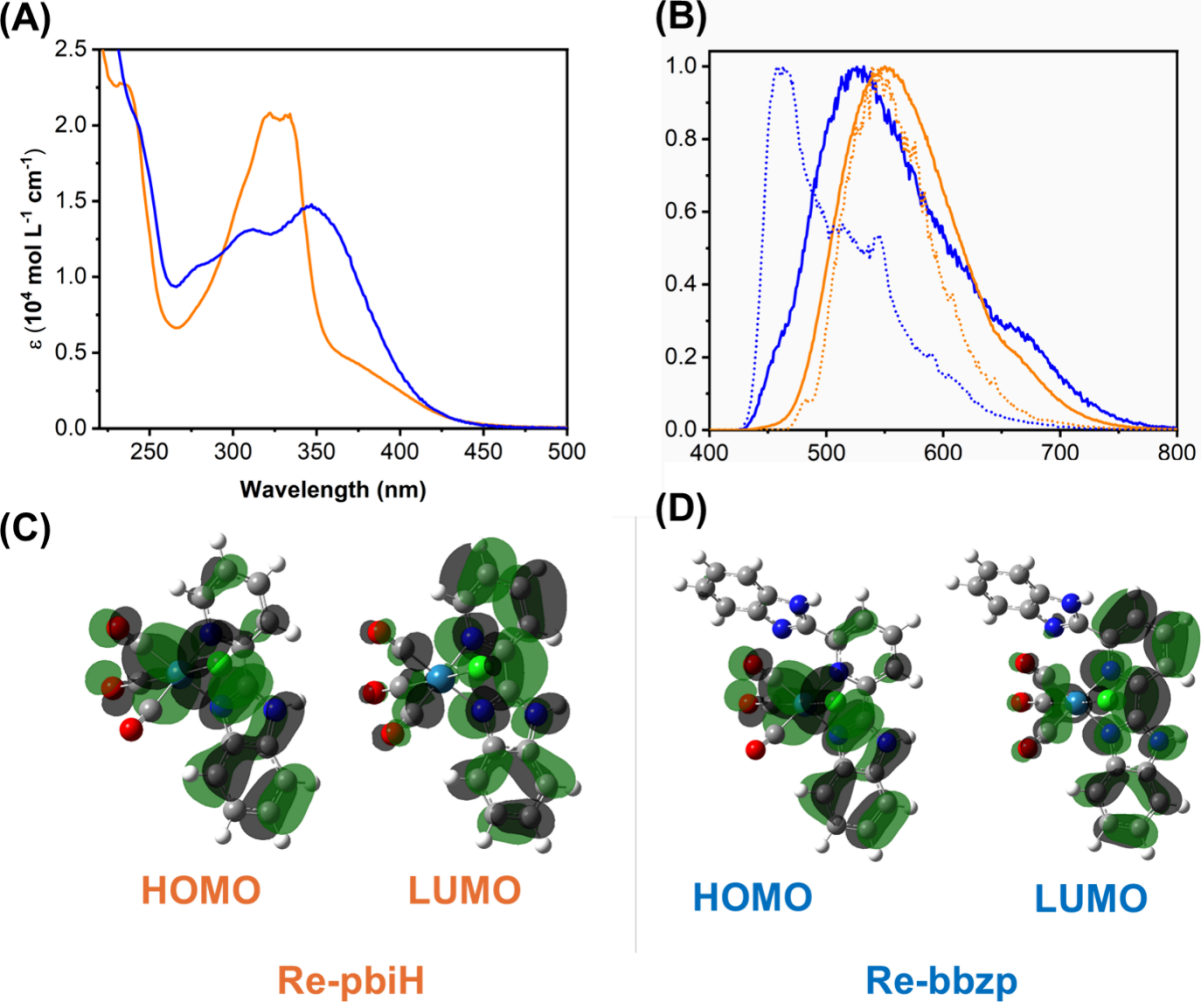
Absorption spectra (A) of the Re-pbiH (orange) and Re-bbzp (blue) in CH_3_CN at 298 K as well as these emission spectra (B) in CH_3_CN at 298 K (solid lines) and butyronitrile at 77 K (dashed lines); λ_exc_ = 375 nm. Surface contours of the HOMO and LUMO orbitals for the Re-pbiH (C) and Re-bbzp (D) complexes.

To gain a more detailed understanding of the lowest-lying excited states of these complexes, TD-DFT calculations were performed using CH_3_CN as the solvent (dielectric constant = 37.5). The first 30 calculated transitions were compared to the corresponding experimental data (Figures S3 and S4). The calculated spectra show good agreement with the experimental results. The main electronic transitions with their respective oscillator strengths are summarized in Tables S1 and S2. The calculations indicate that the lowest absorption bands for both complexes originate mainly from the HOMO–LUMO transitions, the contours of which are displayed in Figure 3c,d. For both complexes, the HOMO is primarily centered on the d orbitals of the Re­(I) center, with some contribution from the π orbitals of the imidazole ligands. The LUMO is mainly localized on the π* orbitals of the pbiH and bbzp ligands, confirming their mixed MLCT/IL character.

Steady-state emission measurements were performed to investigate the excited-state properties of the complexes in CH_3_CN at 298 K and in glassy butyronitrile at 77 K (Figure 3b). The emission spectra at room temperature displayed large Stokes shifts, typical of triplet-emitting states. The emission bands for both complexes are broad and unstructured, as expected for ^3^MLCT emitters, and resemble those of other Re­(I) tricarbonyl polypyridyl complexes.^33–36^ In glassy medium at 77 K, both emission bands undergo a blue shift, which is more pronounced for Re-bbzp than for Re-pbiH, consistent with restricted outer-sphere reorganization that strongly influences ^3^MLCT emitters.^37,38^

Time-resolved emission measurements at 298 K and in CH_3_CN (Figure S4) reveal a relatively short (τ <7 ns) monoexponential decay for Re-bbzp, while for Re-pbiH, a double-exponential decay is observed with longer lifetimes (τ_1_ = 42 ns, 54% and τ_2_ = 466 ns, 46%). The shorter 7 ns lifetime for Re-bbzp agrees well with the smaller emission quantum yield observed for this complex when compared with that for Re-pbiH (Table 1). Furthermore, the photophysical data are also compared to those of the well-known *fac*-[Re­(CO)_3_(bpy)­Cl] (Re-bpy) complex. The decrease in τ and Φ_em_ indicates that the additional pendant benzimidazolyl group in the bbzp ligand promotes nonradiative decay. Further evidence for that is also found in an O_2_-saturated medium, in which the singlet oxygen formation yield (Φ_Δ_) by Re-pbiH is 6-fold higher than that for Re-bbzp.

**Table 1:** Photophysical Properties in CH_3_CN of the Studied Complexes (λ_exc_ = 375 nm)

complexes	λ_max_^abs^ (nm)	ϕ_em_	ϕ_Δ_	τ (ns)^a^	*k*_r_ (×10^4^ s^–1^)	*k*_nr_ (×10^6^ s^–1^)	λ_max_^em^(298 K)
Re-pbiH	320, 375	0.0141	0.30	42 (54%)	33.6^b^	23.4^b^	550 nm
				466 (46%)			
Re-bbzp	300, 360	0.0007	0.05	∼7.0	10.0	142.7	520 nm
Re-bpy	370	0.0058		29.7	66.7	110	590 nm

a Photoluminescence lifetimes probed at the emission maxima.

b Calculated considering the faster decay.

The photophysics of Re-pbiH however seems to be more complex as two distinct excited-state relaxation mechanisms were present, and the excited state was less sensitive to solvent or temperature. The biexponential decay (τ_1_ = 42 ns and τ_2_ = 466 ns) that was evident when the excited state was probed at the emission maximum was monoexponential (τ_1_ = 49 ns) when the excited state was instead probed at longer wavelengths (Figure S5). Moreover, when an excess of acid was added to prevent deprotonation of the Re-pbiH complex, a monoexponential decay was observed along with a red-shifted emission spectrum (Figure S5). The excited-state lifetime with the added acid (τ = 47 ns) was very similar to the fast decay observed for Re-pbiH in CH_3_CN (τ = 42 ns). Hence, these data provide clear evidence for the existence of an acid–base equilibrium in acetonitrile where both the protonated form and the conjugate base are emissive. Therefore, the double-exponential decay observed for the Re-pbiH in CH_3_CN is tentatively assigned to emission from both the protonated and the deprotonated species.

### Electrochemical Properties

The investigated complexes were characterized in an acetonitrile solution with 0.1 M TBAPF_6_ as the supporting electrolyte (Figure 4). At cathodic potentials, Re-pbiH exhibited two close reduction peaks, *Ep* = −2.4 V and *Ep* = −2.5 V vs. Fc^+^/Fc, similar to those observed for Re-bbzp at slightly more negative potentials *Ep* = −2.5 V and −2.7 V vs. Fc^+^/Fc. These observations align with previous literature reports,^39–42^ which have shown that tricarbonyl rhenium complexes typically undergo two sequential reduction processes.

**Figure 4:**
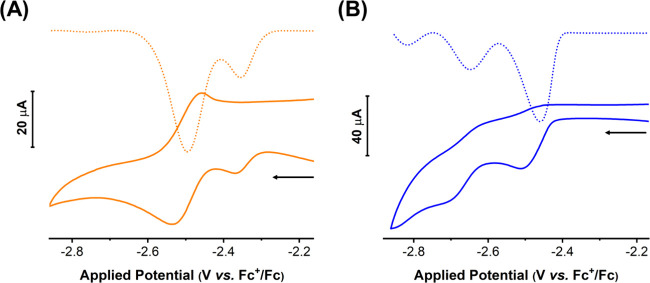
Cyclic voltammograms recorded, with a scan rate of 100 mV s ^–1^ (solid line) and square wave voltammograms measured at a frequency of 50 Hz (dashed line) for Re-pbiH (A) and Re-bbzp (B) complexes in argon-saturated 0.1 M TBAPF_6_/CH_3_CN. The arrow indicates the scan directions.

Square wave voltammetry (SWV) was used to elucidate the number of electrons involved in each reduction peak (Figure S6). According to Lovric’s study, the number of electrons involved in an irreversible process can be calculated by the linear relationship between the peak potential (*E_p_*) versus log f (*f* = applied frequency)*.* ^43,44^ The angular coefficient ( $\frac{\Delta E}{\Delta \log f}$ ) corresponds to Equation S2, which was used to estimate the *n* value (where α = transfer coefficient and *n* = number of electrons). Assuming α = 1.0, an *n* value was obtained for each process, showing that the first reduction peak involved *n* = 2, while *n* = 1 for the second reduction (Figure S6). This behavior agrees well with the literature, where the first peak is attributed to ligand-based reduction with chloride labilization, resulting in *n* = 2 in the Lovric’s equation due to the involvement of one electron and anionic charge labilization.^45^ The second reduction peak has previously been assigned to a metal reduction, with only one electron participating (*n* = 1).^45–47^ To validate the Lovric approach, the same experiment and analysis was performed on the well-known *fac*-[Re­(CO)_3_(bpy)­Cl] (Figure S7), which revealed *n* = 2 and *n* = 1 for the first and second reduction, respectively.

### Photodriven CO_2_ Reduction

Photoreduction of carbon dioxide was evaluated by employing a Xe lamp with a 370 nm long-pass filter as the white light excitation source. With λ_exc_ >370 nm, only the photocatalyst absorbs light and not the electron donor (see Re-bbzp, Re-pbiH, and BIH overlapped absorption spectrum in Figure S8). Solutions containing 0.03 mmol L^–1^ photocatalyst and an excess of 6.7 mmol L^–1^ BIH as a donor in acetonitrile were irradiated under a CO_2_ atmosphere. The gaseous products were monitored by gas chromatography with samples being collected from the headspace every 30 min interval for 300 min.

In the present conditions, CO was identified as the sole CO_2_ reduction product. Control experiments carried out under an argon atmosphere, in the absence of the photocatalysts, or in the dark did not yield any CO. In Figure 5, the CO evolution as a function of the irradiation time for both complexes is shown. The Re-bbzp complex exhibited significantly better performance, reaching a turnover number (TON) of 45 ± 2 after 4.5 h of irradiation, while Re-pbiH exhibits a maximum TON of 8 ± 1 in the same reaction conditions. Long-term (20 h) CO evolution experiments for Re-bbzp confirm that the plateau is achieved around 4.5 h of photocatalysis, Figure S9.

**Figure 5:**
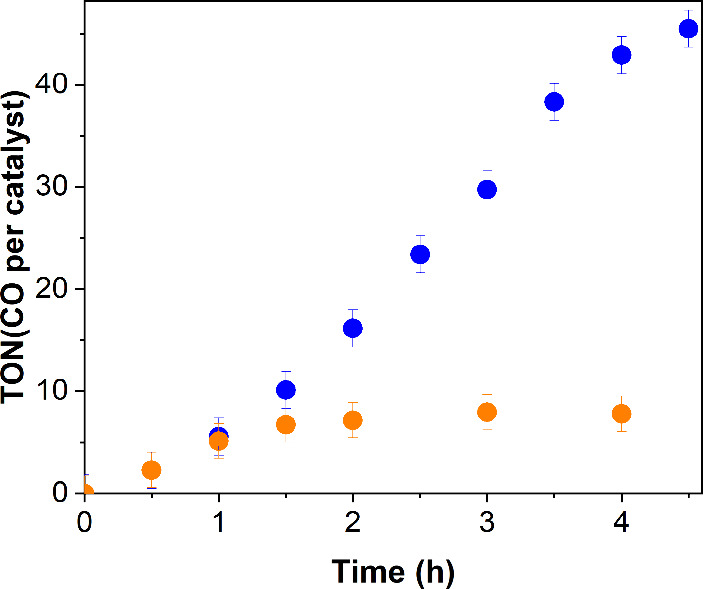
Turnover number (TON) for CO formation photocatalyzed by Re-pbiH (orange) and Re-bbzp (blue) complexes. Experiments were performed in CO_2_-saturated 10 mL CH_3_CN solutions containing 6.7 mmol L^–1^ of BIH and 0.03 mmol L^–1^ of photocatalysts (λ >370 nm).

Therefore, the introduction of a second benzimidazolyl group onto the Re catalyst directly impacts the CO_2_ reduction efficiency, resulting in a 5.6-fold increase in the level of CO evolution. On the other hand, photocatalytic experiments carried out with different concentrations of added water (0.001, 0.002, and 0.020%) for the Re-bbzp complex revealed enhanced catalyst deactivation as the water concentration increased (Figure 6a). Moreover, when BIH was used as a donor in the presence of triethanolamine (CH_3_CN/TEOA 5:1 v/v), a decrease in the Re-pbiH catalysis was observed for the reduction of CO_2_ with H_2_ being formed instead. For the more optimal photocatalyst, Re-bbzp, a complete loss of CO_2_ reduction was observed under this condition over the 3 h time interval of the experiment (Table S3). We hypothesize that protonation of an N–H site on the additional benzimidazolyl moiety on the bbzp ligand is a key step to promote the CO_2_ reduction, as its proximity to the metal center favors the stabilization of key reaction intermediates. The addition of a second proton acceptor (water or TEOA) introduces a competing pathway for N–H protonation, thereby inhibiting the CO_2_ photocatalytic activity.

**Figure 6:**
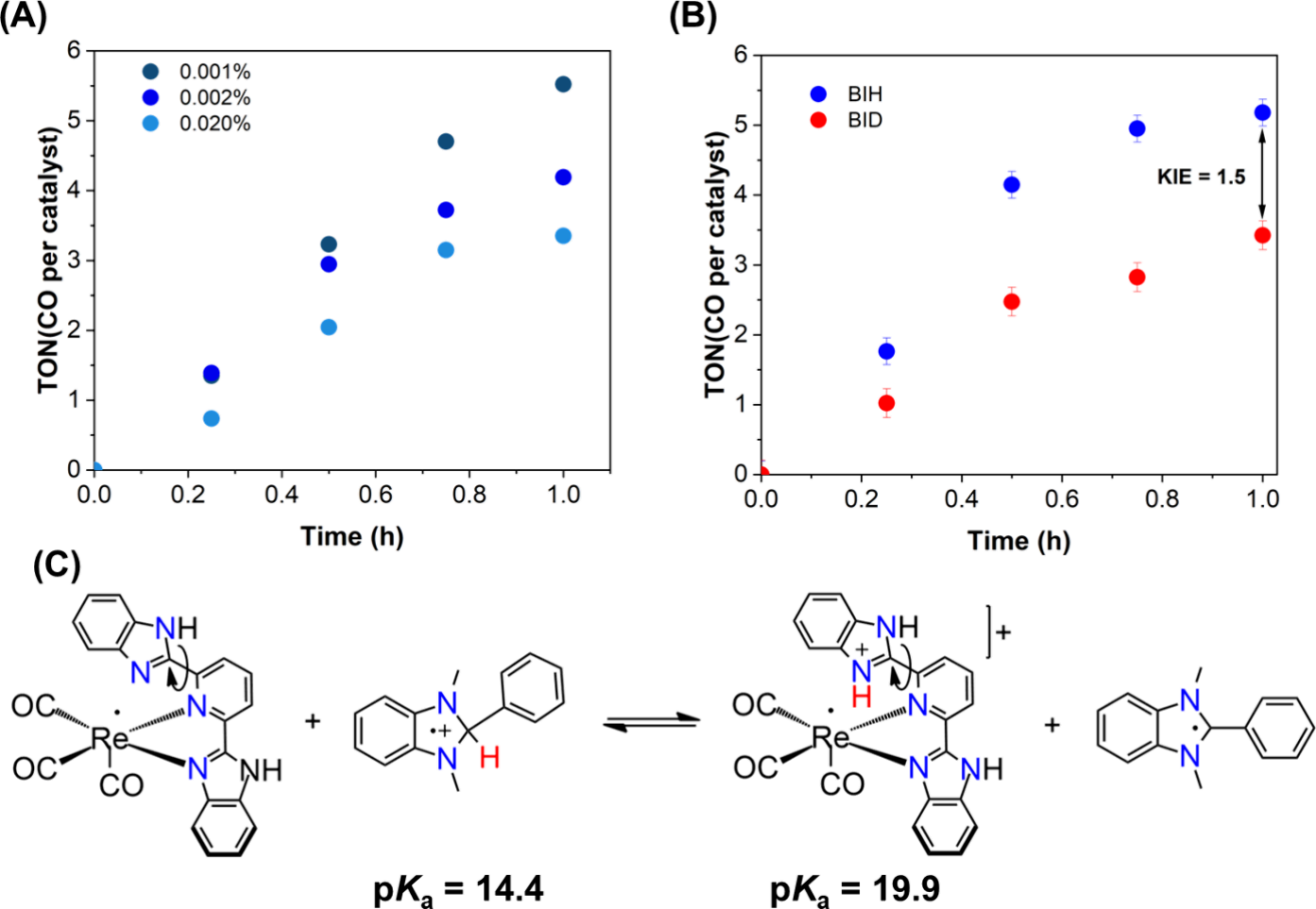
(a) Turnover number (TON) for CO formation mediated by Re-bbzp in CO_2_-saturated CH_3_CN containing BIH (6.7 mmol L^–1^) and Re-bbzp (0.03 mmol L^–1^) and the indicated H_2_O concentrations (λ_exc_ >370 nm). (b) Turnover number (TON) for CO formation using BIH and BID from which a kinetic isotope effect was extracted (KIE = 1.5). (c) Isodesmic reaction employed to estimate the pK_a_ of the protonated reduced Re-bbzp intermediate, using BIH^·^+^^ as the reference acid.

### Ligand-Assisted Proton Transfer

To investigate this possibility, an isodesmic reaction was considered between the oxidized form of BIH (BIH·^+^) and the first reduced Re-bbzp species formed following chloride labilization (see the next section). Isodesmic reactions enable accurate theoretical pK_a_ determinations by comparing a target acid to a reference acid with a known pK_a_.^48^ Here, the reported pK_a_ value of BIH·^+^ (14.4 in acetonitrile) was employed as the reference.^49^ The calculated pK_a_ of 19.9 for the first reduced and protonated intermediate supports the feasibility of protonation at a third amine site during the catalytic pathway as shown in Figure 6c and is also compatible to the effective acidity of CO_2_-derived species in acetonitrile medium (pK_a_ ≈ 23–25).^50,51^ Density functional theory (DFT) geometry optimizations further reveal that this protonated N–H site is oriented toward the CO_2_ molecule coordinated to the metal center, enabling the formation of a stabilizing hydrogen bond between the ligand and the CO_2_ adduct (Figure S10). Such an interaction is expected to facilitate CO_2_ activation during the reduction process and aligns well with the conclusion by Mukherjee and Siewert that proton relays near the axial positions have beneficial effects on the catalytic activity of Re­(I) complexes.^52^

To experimentally probe proton transfer from the coordinated bbzp ligand to the CO_2_ adduct, photocatalytic experiments were conducted using BID as the electron donor (Figure 6b), in which the labile proton of BIH is replaced by deuterium (see Figure S11 for ^1^H NMR characterization). Photocatalysis performed with BID exhibits a kinetic isotope effect (KIE) of 1.5 in relation to that with BIH, indicating that proton transfer from BIH to the reduced Re-bbzp species is involved in the rate-determining steps of the catalytic cycle.

The proposed ligand-assisted proton transfer mechanism provides a simple explanation for the superior photocatalytic activity of Re-bbzp relative to that of Re-pbiH for CO_2_ reduction. The Re-bbzp achieves optimal photocatalytic performance without the presence of Brønsted bases, which would otherwise compete with ligand protonation and disrupt the ligand-assisted proton relay essential for efficient CO_2_ reduction. To gain further mechanistic insights into the CO_2_ reduction activity of the Re-bbzp complex, spectroelectrochemical FTIR (IR-SEC) and *in situ* FTIR experiments were performed under both Ar and CO_2_ atmospheres.

### Catalytic Pathway Analysis of Re-bbzp Complex

During IR-SEC measurements, the characteristic CO stretching modes (A_1_ and E, molecular symmetry C_3v_)^53^ of Re-bbzp were monitored during potential steps that initiated the first or second reductions (Figure S12). The observed spectral data closely resemble those reported for *fac*-[Re­(CO)_3_(bpy)­Cl] derivatives.^45,54–57^ Figure 7a,b highlights the evolution of the νCO region during the reduction of Re-bbzp under Ar and CO_2_ atmospheres, respectively.

**Figure 7:**
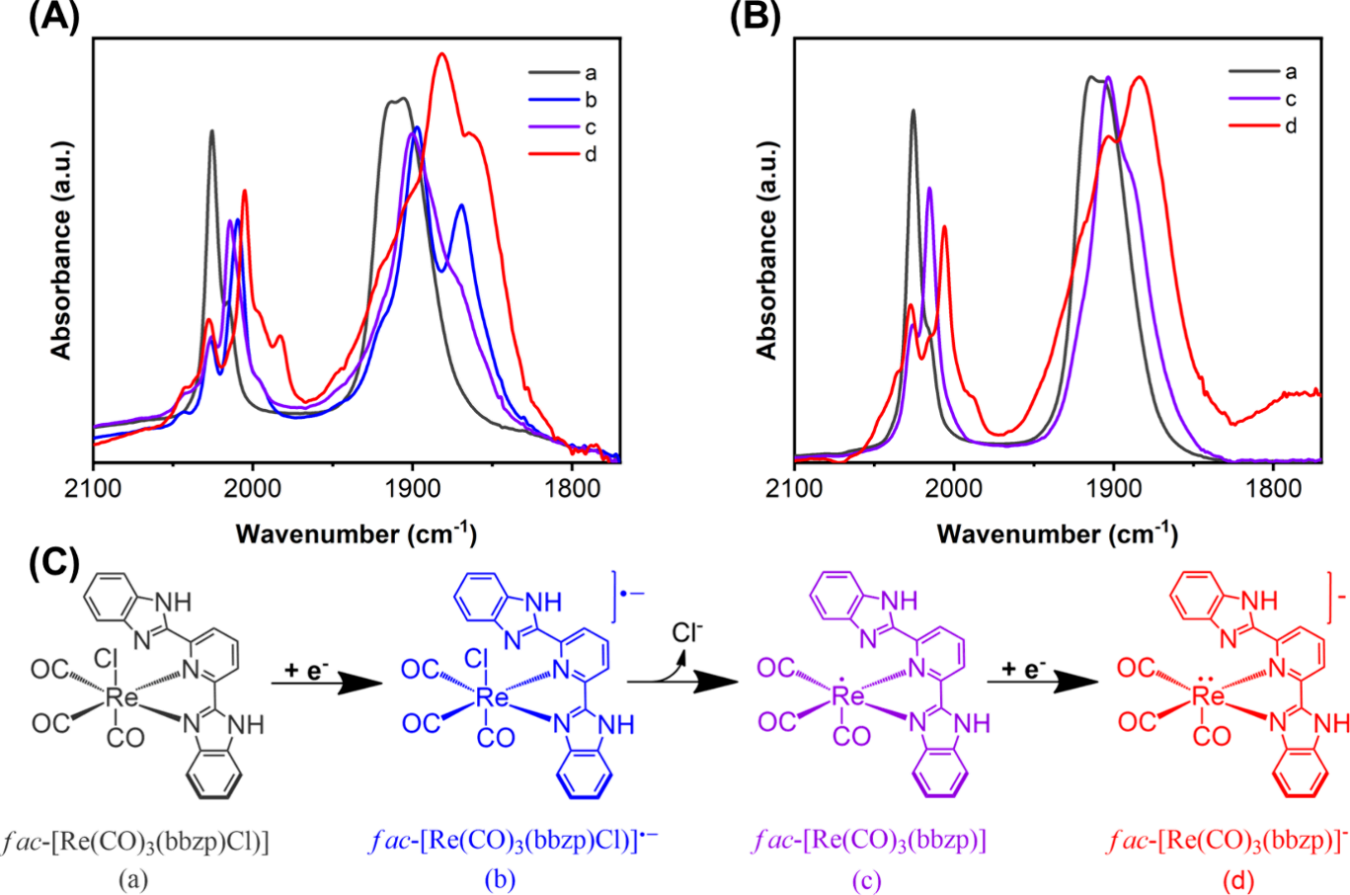
Infrared spectroelectrochemistry (IR-SEC) of Re-bbzp in 0.1 M TBAPF_6_/CH_3_CN electrolyte for the first (*E*_*p*_ = −2.5 V vs. Fc^+^/Fc) and second (*E*_*p*_ = −2.7 V vs Fc^+^/Fc) electron reduction process under Ar (A) and CO_2_ (B) atmosphere. (C) Proposed reduction pathway for Re-bbzp complex (a) to species (b), (c), and (d) color-coded to the spectral assignments in panels (A) and (B).

Upon the first reduction, the complex *fac*-[Re­(CO)_3_(bbzp)­Cl] (a) gives rise to new νCO bands at 2009, 1897, and 1869 cm^–1^, which are assigned to the reduced intermediate *fac*-[Re­(CO)_3_(bbzp)­Cl]^−^· (b). In this species, the added electron is primarily localized on the π* orbitals of the diimine ligand, increasing the electron density at the Re­(I) center and weakening the CO bonds, resulting in a νCO shift to lower frequencies.^57–60^ After approximately 60 s at the same applied potential, species (b) gradually convert into species (c), *fac*-[Re­(CO)_3_(bbzp)], characterized by new bands at 2014 and 1900 cm^–1^ (Figure S13). This transformation is attributed to chloride labilization, yielding a pentacoordinated, one electron-reduced species. The corresponding νCO shift to higher frequencies reflects a decrease in electron density at the metal center following chloride dissociation.^3,46,61,62^ This pathway is further supported by Lovric’s analysis, which indicates *n* = 2 for the first reduction process, consistent with the involvement of two charged species. Notably, species (c) remains stable over extended times at this potential.

A potential step beyond the second reduction potential led to the appearance of additional νCO bands centered at 2005, 1983, 1882, and 1867 cm^–1^, assigned to the two-electron-reduced species *fac*-[Re­(CO)_3_(bbzp)]^−^(d). The second reduction increases electron density on the Re center, leading to a νCO shift to lower frequencies.^61^ The proposed electrochemical reduction pathway is summarized in Figure 7c. Comparative IR-SEC experiments performed under a CO_2_ atmosphere revealed a similar reaction chemistry. Eventual differences in the peak shapes when compared to those under the Ar atmosphere are attributed to changes in the relative concentration of the species as the different potentials are applied and, therefore, are not considered in the analysis. Different from what is observed under argon, in the presence of CO_2_, the intermediate *fac*-[Re­(CO)_3_(bbzp)­Cl]^−^· (b) was not observed (Figure S14). This suggests that the presence of CO_2_ accelerates chloride labilization, likely because it is a better π* acceptor ligand than Cl^–^, favoring the rapid formation of species (c), which is subsequently reduced to species (d).

*In situ* FTIR experiments (Figure 8) conducted during photocatalytic CO_2_ reduction reveal spectral changes closely matching those observed by IR-SEC, confirming the formation of analogous intermediates under photochemical conditions (see the direct comparison in Figure S15).

**Figure 8:**
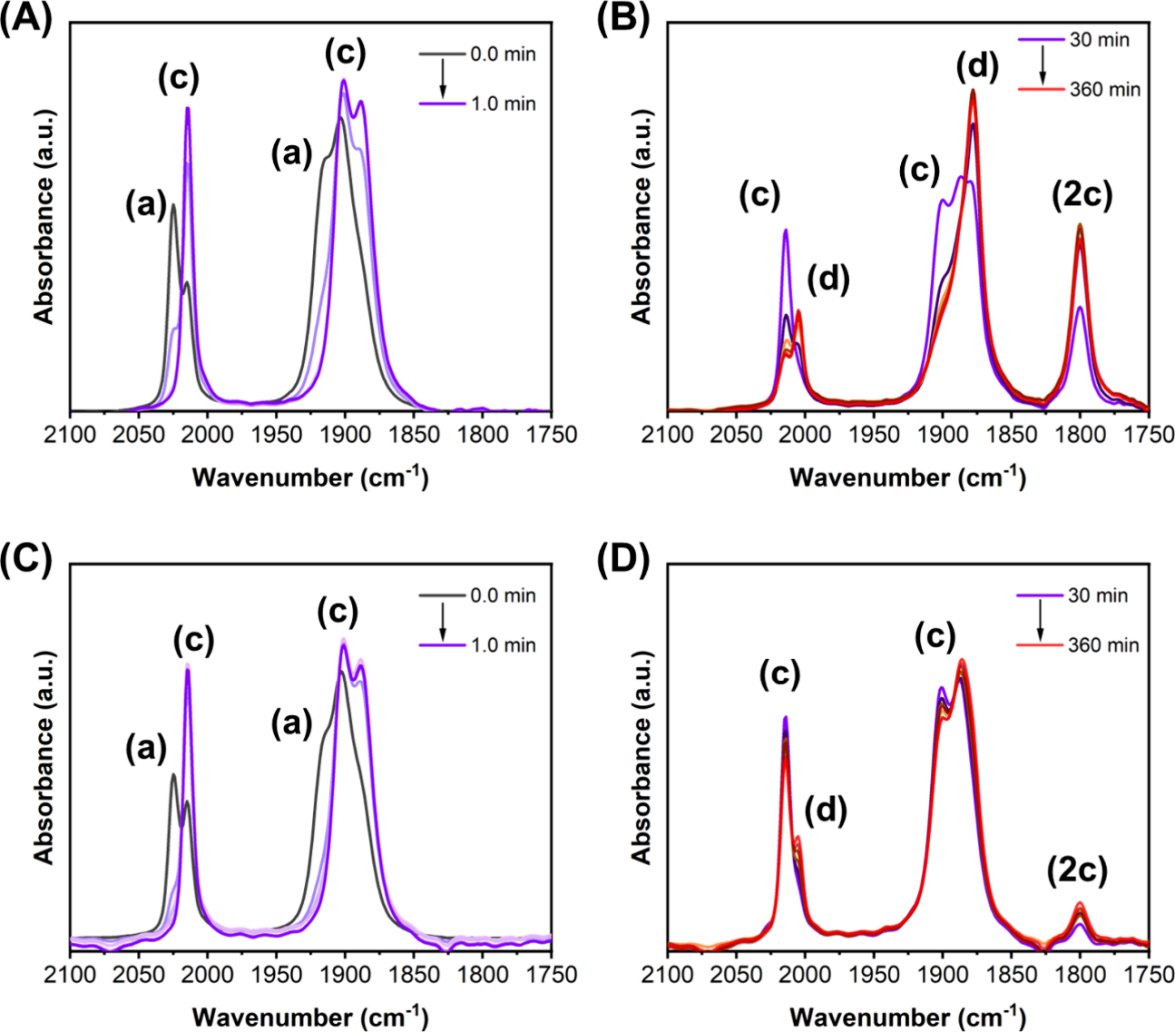
(A–D) *In situ* FTIR spectra measured over the indicated photolysis time for Re-bbzp in Ar-saturated (a and b) and CO_2_-saturated (c and d) CH_3_CN solutions containing excess BIH.

Under argon atmosphere (Figure 8a,b), *fac*-[Re­(CO)_3_(bbzp)] (c) forms rapidly (<1 min) without detectable accumulation of species *fac*-[Re­(CO)_3_(bbzp)­Cl]^−^· (b). Prolonged irradiation leads to the gradual formation of the two-electron-reduced *fac*-[Re­(CO)_3_(bbzp)]^−^ (d). Concurrently, an additional band at 1800 cm^–1^ appears, attributed to a μ-CO-bridged dimeric species [Re­(CO)_3_(bbzp)]_2_(**2c**).

The formation of dimeric species during photocatalytic CO_2_ reduction by *fac*-[Re­(CO)_3_(NN)­Cl]-type complexes (where NN = 2,2′-bipyridine derivatives) is a well-established and extensively documented deactivation pathway. Kubiak and Fujita independently isolated and spectroscopically characterized Re–Re bonded dimers [Re­(CO)_3_(NN)]_2_, which display exclusively terminal CO stretching modes in the ∼1840–2000 cm^–1^ region and a characteristic absorption near 700 nm.^3,63,64^ In contrast, the *in situ* FTIR spectra of Re-bbzp show an additional band at 1800 cm^–1^ without the presence of a new visible absorption band (Figure S16). This spectral signature is assigned to a μ-CO-bridged dimeric species rather than a metal–metal-bonded Re–Re dimer. Bridging CO ligands are well known to exhibit IR absorptions in the 1700–1850 cm^–1^ range, as reported for μ-CO-bridged cobalt and chromium carbonyl complexes.^65–67^ Hence, the pendant benzimidazolyl group in the bbzp ligand appears to provide steric hindrance for the Re–Re bond formation.

Under a CO_2_ atmosphere, the same intermediates were observed but with markedly different kinetics. Species *fac*-[Re­(CO)_3_(bbzp)] (c) persisted for significantly longer times, while formation of species *fac*-[Re­(CO)_3_(bbzp)]^−^(d) was suppressed relative to those measured under an argon atmosphere, supporting a catalytic mechanism in which the two-electron-reduced *fac*-[Re­(CO)_3_(bbzp)]^−^ is the active species for CO_2_ reduction. Moreover, the μ-CO-bridged dimer band at 1800 cm^–1^ was substantially less intense, indicating that the CO_2_ inhibits dimer formation and stabilizes catalytically relevant intermediates.

Figure S17 displays the kinetic evolution of the monitored species by tracking fixed wavenumbers at 2025, 2015, 2005, and 1800 cm^–1^, corresponding to species (a), (c), (d), and (2c), respectively. Within the first minute of irradiation, complex *fac*-[Re­(CO)_3_(bbzp)­Cl] (a) is fully consumed and converted predominantly into *fac*-[Re­(CO)_3_(bbzp)] (c), with partial formation of the two-electron-reduced species *fac*-[Re­(CO)_3_(bbzp)]^−^(d). Upon prolonged irradiation, *fac*-[Re­(CO)_3_(bbzp)] (c) is progressively consumed, accompanied by the growth of the μ-CO-bridged dimeric species [Re­(CO)_3_(bbzp)]_2_(**2c**) and continued formation of *fac*-[Re­(CO)_3_(bbzp)]^−^(d). Notably, the formation rate of *fac*-[Re­(CO)_3_(bbzp)]^−^(d) decreases as the concentration of [Re­(CO)_3_(bbzp)]_2_(**2c**) increases, indicating that dimerization occurs in parallel with the two-electron reduction of *fac*-[Re­(CO)_3_(bbzp)] (c). Under a CO_2_ atmosphere, distinct kinetics were observed. In this case, *fac*-[Re­(CO)_3_(bbzp)] (c) was not fully consumed, while the formation of *fac*-[Re­(CO)_3_(bbzp)]^−^(d) and [Re­(CO)_3_(bbzp)]_2_(**2c**) occurred as competing processes. Since no subsequent decrease in the concentration of [Re­(CO)_3_(bbzp)]_2_(**2c**) was observed, this pathway was identified as a photocatalytically inactive deactivation route, arising from the coupling of two equivalents of *fac*-[Re­(CO)_3_(bbzp)] (c). Moreover, we propose that after CO formation and release of H_2_O, the resulting Re species is rapidly reduced by BIH, regenerating *fac*-[Re­(CO)_3_(bbzp)] (c). This regeneration process is supported by the persistent presence of species (c) throughout the experiments conducted under a CO_2_ atmosphere.

Finally, based on these findings, a photocatalytic cycle for Re-bbzp using BIH as an electron donor is proposed (Figure 9), incorporating intermediates identified by both IR-SEC and *in situ* FTIR. Protonation of the Re-bbzp framework by BIH^·^+^^ was supported by the observed kinetic isotope effect (KIE = 1.5) when BID is used as an electron donor as discussed previously. We assign the active species for reaction with CO_2_ to the two-electron-reduced species that was observed under an argon atmosphere yet was absent under a CO_2_ atmosphere. The formation of the μ-CO-bridged dimeric species contributes to the photocatalytic deactivation, and it occurs as a side reaction from the one electron-reduced species as demonstrated by the *in situ* FTIR experiments.

**Figure 9:**
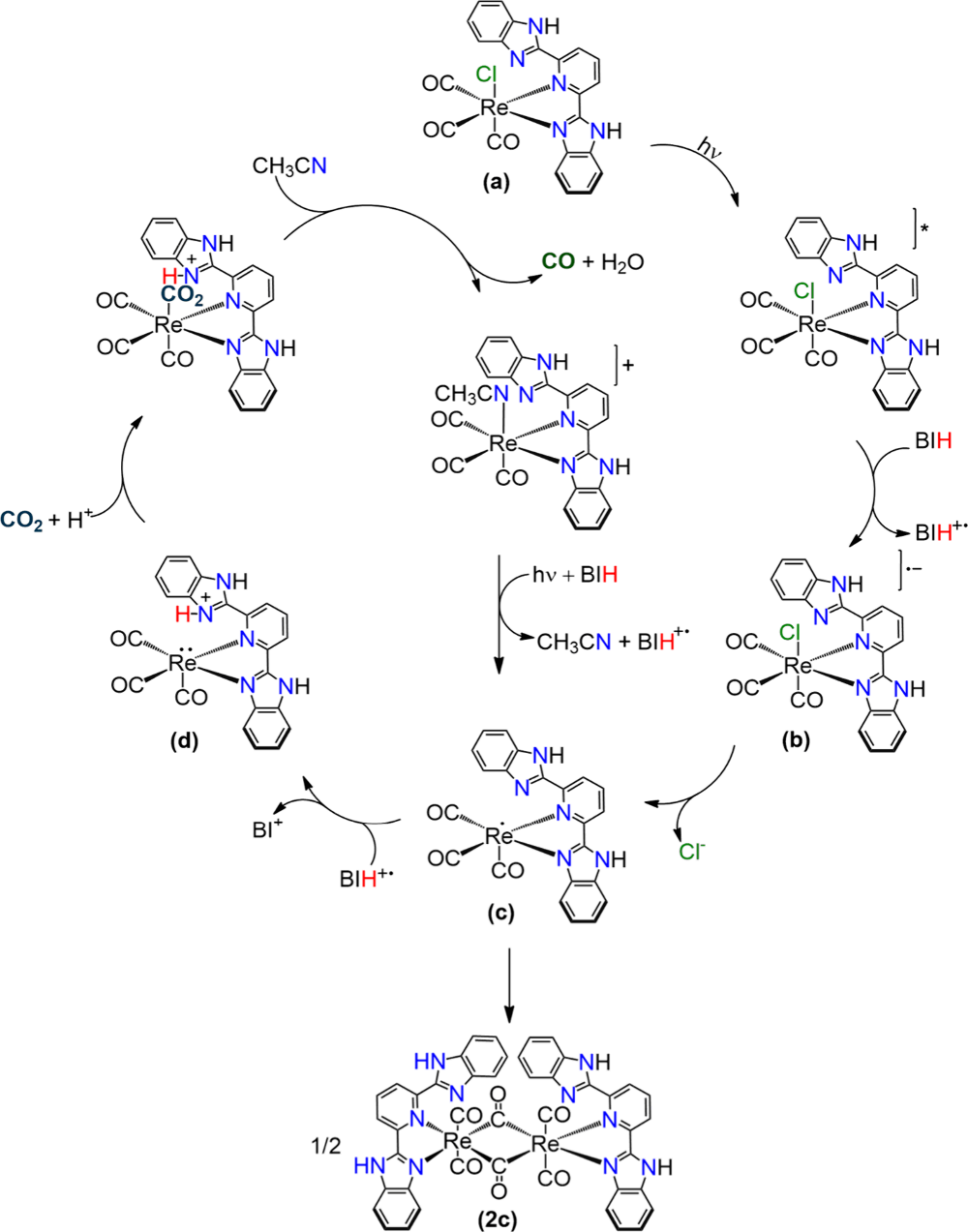
Proposed photocatalytic cycle for Re-bbzp using BIH as an electron donor in acetonitrile.

## Conclusions

In this study, the photocatalytic CO_2_-to-CO reduction behavior of two Re­(I) tricarbonyl complexes bearing imidazole–pyridine ligands, *fac*-[Re­(CO)_3_(pbiH)­Cl] and *fac*-[Re­(CO)_3_(bbzp)­Cl], was investigated with the aim of elucidating the role of the ligand structure on the catalytic activity and mechanism. While both complexes display comparable photophysical and electrochemical properties, the incorporation of an additional benzimidazole unit in Re-bbzp led to a pronounced enhancement in photocatalytic performance relative to Re-pbiH. A combination of IR spectroelectrochemistry and *in situ* FTIR spectroscopy under photocatalytic conditions enabled the direct identification of key intermediates involved in the catalytic cycle. These studies reveal that chloride labilization occurs rapidly upon reduction, generating a pentacoordinate one electron-reduced Re species that can undergo further reduction to form the two-electron-reduced intermediate (active species toward the CO_2_ reaction). In parallel, dimerization pathways were observed, which act as deactivation channels during photocatalysis. Importantly, the *in situ* FTIR data indicate that dimer formation proceeds predominantly through μ-CO-bridged species rather than the well-known Re–Re metal–metal-bonded dimers reported for related Re­(I) tricarbonyl complexes.

The superior activity of Re-bbzp was rationalized by its ability to function as a ligand-based proton-responsive system. Kinetic isotope effect measurements using a deuterated BIH donor support the involvement of proton transfer steps during the catalytic cycle, consistent with a mechanism in which protonation of the pendant benzimidazole unit facilitates the CO_2_ activation. The sensitivity of catalytic performance to the presence of external Brønsted bases further underscores the critical role of ligand protonation dynamics in governing reactivity. Overall, these results demonstrate that the integration of proton-responsive functionalities into imidazole–pyridine ligands serve as an effective strategy to promote productive catalytic pathways. This mechanistic insight provides a foundation for the rational design of next-generation Re­(I) photocatalysts and related molecular systems for CO_2_ reduction.

## Supplementary Material



## Notes

Experimental details, materials, and methods, including photographs of experimental setup and additional catalytic and mechanistic experiments (PDF)

## References

[1] Hawecker J., Lehn J. M., Ziessel R. (1983). Efficient photochemical reduction
of CO2 to CO by visible light irradiation of systems containing Re­(bipy)­(CO)­3X
or Ru­(bipy) 32+-Co2+ combinations as homogeneous catalysts. Chem. Commun..

[2] Kuramochi Y., Ishitani O., Ishida H. (2018). Reaction mechanisms
of catalytic
photochemical CO2 reduction using Re­(I) and Ru­(II) complexes. Coord. Chem. Rev..

[3] Müller A. V., Faustino L. A., de Oliveira K. T., Patrocinio A. O. T., Polo A. S. (2023). Visible-Light-Driven Photocatalytic
CO2 Reduction by
Re­(I) Photocatalysts with N-Heterocyclic Substituents. ACS Catal..

[4] Takeda H., Ishitani O. (2010). Development of efficient photocatalytic
systems for
CO2 reduction using mononuclear and multinuclear metal complexes based
on mechanistic studies. Coord. Chem. Rev..

[5] Sahara G., Ishitani O. (2015). Efficient photocatalysts for CO2
reduction. Inorg. Chem..

[6] Silva G. N., Faustino L. A., Nascimento L. L., Lopes O. F., Patrocinio A. O. T. (2024). Visible
light-driven CO2 photoreduction by a Re­(I) complex immobilized onto
CuO/Nb2O5 heterojunctions. J. Chem. Phys..

[7] Faustino L. A., Hora Machado A. E., Patrocinio A. O. T. (2018). Photochemistry of fac-[Re­(CO)­3­(dcbH2)­(trans-stpy)]+:
New Insights on the Isomerization Mechanism of Coordinated Stilbene-like
Ligands. Inorg. Chem..

[8] Müller A. V., Ahmad S., Sirlin J. T., Ertem M. Z., Polyansky D. E., Grills D. C., Meyer G. J., Sampaio R. N., Concepcion J. J. (2024). Reduction
of CO to Methanol with Recyclable Organic Hydrides. J. Am. Chem. Soc..

[9] Crudo N. R., Sonea A., Karn L. M., Leznoff D. B., Warren J. J. (2025). Modifying
the Rate of Rhenium (Diimine)-Mediated Electrochemical Carbon Dioxide
Reduction via the Addition of a Redox-Active Functional Group Near
the Active Site. ACS Catal..

[10] Abudayyeh A. M., De Kreijger S., Grau S., Eryılmaz E., Robeyns K., Ertem M. Z., Llobet A., Elias B., Troian-Gautier L. (2026). Enhanced Electrocatalytic
and Selective CO2-to-CO Reduction
by a Rhenium (I) Complex Bearing 6, 6′-Substituted 2, 2′-Bipyridines. ACS Catal..

[11] Qiu L. Q., Chen K. H., Yang Z. W., Ren F. Y., He L. N. (2021). Prolonging
the Triplet State Lifetimes of Rhenium Complexes with Imidazole-Pyridine
Framework for Efficient CO2 Photoreduction. Chem.—Eur. J..

[12] Kurz P., Probst B., Spingler B., Alberto R. (2006). Ligand Variations in
[ReX­(diimine)­(CO)­3] Complexes: Effects on Photocatalytic CO2 Reduction. Eur. J. Inorg. Chem..

[100] Sousa S. F., Sampaio R. N., Barbosa
Neto N. M., Machado A. E. H., Patrocinio A. O. T. (2014). The
photophysics of fac-[Re­(CO)­3­(NN)­(bpa)]+
complexes: a theoretical/experimental study. Photochem Photobiol Sci.

[13] Tamaki Y., Koike K., Morimoto T., Ishitani O. (2013). Substantial improvement
in the efficiency and durability of a photocatalyst for carbon dioxide
reduction using a benzoimidazole derivative as an electron donor. J. Catal..

[14] Chow Y. L., Danen W. C., Nelsen S. F., Rosenblatt D. H. (1978). Nonaromatic
aminium radicals. Chem. Rev..

[15] Manke A.-M., Geisel K., Fetzer A., Kurz P. (2014). A water-soluble tin­(IV)
porphyrin as a bioinspired photosensitiser for light-driven proton-reduction. Phys. Chem. Chem. Phys..

[16] Hasegawa E., Takizawa S., Seida T., Yamaguchi A., Yamaguchi N., Chiba N., Takahashi T., Ikeda H., Akiyama K. (2006). Photoinduced electron-transfer systems
consisting of electron-donating pyrenes or anthracenes and benzimidazolines
for reductive transformation of carbonyl compounds. Tetrahedron.

[17] Rotundo L., Grills D. C., Gobetto R., Priola E., Nervi C., Polyansky D. E., Fujita E. (2021). Photochemical CO2 Reduction Using
Rhenium (I) Tricarbonyl Complexes with Bipyridyl-Type Ligands with
and without Second Coordination Sphere Effects. ChemPhotoChem..

[18] Agarwal J., Fujita E., Schaefer H. F., Muckerman J. T. (2012). Mechanisms
for CO production from CO2 using reduced rhenium tricarbonyl catalysts. J. Am. Chem. Soc..

[19] Gibson D. H., Yin X., He H., Mashuta M. S. (2003). Synthesis and Reactions of fac-[Re
(dmbpy)­(CO) 3X]­(dmbpy= 4, 4 ‘-Dimethyl-2, 2 ‘-bipyridine;
X= COOH, CHO) and Their Derivatives. J. Organomet.
Chem..

[20] Costentin C., Drouet S., Robert M., Savéant J.-M. (2012). A local
proton source enhances CO2 electroreduction to CO by a molecular Fe
catalyst. Science.

[21] Franco F., Cometto C., Vallana F. F., Sordello F., Priola E., Minero C., Nervi C., Gobetto R. (2014). A local proton source
in a [Mn­(bpy-R)­(CO)­3Br]-type redox catalyst enables CO2 reduction
even in the absence of Brønsted acids. Chem. Commun..

[22] Agarwal J., Shaw T. W., Schaefer H. F., Bocarsly A. B. (2015). Design
of a catalytic active site for electrochemical CO2 reduction with
Mn (I)-tricarbonyl species. Inorg. Chem..

[23] Ngo K. T., McKinnon M., Mahanti B., Narayanan R., Grills D. C., Ertem M. Z., Rochford J. (2017). Turning on the protonation-first
pathway for electrocatalytic CO2 reduction by manganese bipyridyl
tricarbonyl complexes. J. Am. Chem. Soc..

[24] Barlow J. M., Gupta N., Glusac K. D., Tiede D. M., Kaphan D. M. (2024). Proton-Responsive
Ligands Promote CO2 Capture and Accelerate Catalytic CO2/HCO2–Interconversion. Inorg. Chem..

[25] Wilting A., Stolper T., Mata R. A., Siewert I. (2017). Dinuclear Rhenium Complex
with a Proton Responsive Ligand as a Redox Catalyst for the Electrochemical
CO(2) Reduction. Inorg. Chem..

[26] Wilting A., Siewert I. (2018). A Dinculear Rhenium Complex with a Proton Responsive
Ligand in the Electrochemical-Driven CO2-Reduction Catalysis. ChemistrySelect.

[27] Du J. P., Wilting A., Siewert I. (2019). Are Two Metal
Ions Better than One?
Mono-and Binuclear α-Diimine-Re (CO) 3 Complexes with Proton-Responsive
Ligands in CO2 Reduction Catalysis. Eur. J.
Inorg. Chem..

[28] Hanson S. S., Warren J. J. (2018). Syntheses, characterization, and electrochemical behavior
of alkylated 2-(2′-quinolylbenzimidazole) complexes of rhenium
(I). Can. J. Chem..

[29] Sinha S., Sonea A., Gibbs C. A., Warren J. J. (2020). Heterogeneous aqueous
CO(2) reduction by rhenium­(i) tricarbonyl diimine complexes with a
non-chelating pendant pyridyl group. Dalton
Trans..

[30] Sinha S., Berdichevsky E. K., Warren J. J. (2017). Electrocatalytic CO2 reduction using
rhenium­(I) complexes with modified 2-(2’-pyridyl)­imidazole
ligands. Inorg. Chim. Acta.

[31] do Carmo, M. E. G. ; de Matos, P. A. ; Maia, P. I. S. ; Machado, A. E. H. ; Beletti, M. E. ; Tsubone, T. M. ; Patrocinio, A. O. T. , The photophysics of Ir­(III) cyclometalated complexes containing the 2-(2-pyridyl)­benzimidazole ancillary ligand: Protonation effect and their potential as specific lysosome probes in cells. J. Photochem. Photobiol., A 2024, 448.

[32] Sato S., Matubara Y., Koike K., Falkenstrom M., Katayama T., Ishibashi Y., Miyasaka H., Taniguchi S., Chosrowjan H., Mataga N., Fukazawa N., Koshihara S., Onda K., Ishitani O. (2012). Photochemistry of fac-[Re­(bpy)­(CO)­3Cl]. Chem.—Eur. J..

[33] Ramos L. D., Sampaio R. N., De Assis F. F., De Oliveira K. T., Homem-De-Mello P., Patrocinio A. O. T., Frin K. P. M. (2016). Contrasting photophysical
properties of rhenium­(i) tricarbonyl complexes having carbazole groups
attached to the polypyridine ligand. Dalton
Trans..

[34] Itokazu M. K., Polo A. S., de Faria D. L. A., Bignozzi C. A., Iha N. Y. M. (2001). Syntheses
and spectroscopic characterization of fac-[Re (CO) 3 (phen)­(L)] PF6,
L= trans-and cis-1, 2-bis (4-pyridyl) ethylene. Inorg. Chim. Acta.

[35] Ramos L. D., da Cruz H. M., Morelli
Frin K. P. (2017). Photophysical properties of rhenium
(I) complexes and photosensitized generation of singlet oxygen. Photochem. Photobiol. Sci..

[36] Mamud J. F., Biazolla G., Marques C. S., Cerchiaro G., de Queiroz T. B., Keppler A. F., Polo A. S. (2021). Z to E
light-activated
isomerization of α-pyridyl-N-arylnitrone ligands sensitized
by rhenium (I) polypyridyl complexes. Inor.
Chim. Acta.

[37] Lees A. J. (1995). The Luminescence
Rigidochromic Effect Exhibited by Organometailic Complexes: Rationale
and Applications. Comments Inorg. Chem..

[38] Patrocinio A. O. T., Brennaman M. K., Meyer T. J., Murakami Iha N. Y. (2010). Excited-state
dynamics in fac- [Re­(CO)­3­(Me4phen)­(L)] +. J.
Phys. Chem. A.

[39] Müller A. V., Wierzba W. M., do Nascimento L. G. A., Concepcion J. J., Nikolaou S., Polyansky D. E., Polo A. S. (2025). Tuning the Photocatalytic
CO2 Reduction through para-Substituents in Bipyridyl Rhenium Complexes. Artif. photosynth..

[40] Grice K. A., Kubiak C. P. (2014). Recent Studies of Rhenium and Manganese Bipyridine
Carbonyl Catalysts for the Electrochemical Reduction of CO2. Adv. Inorg. Chem..

[41] Roell S. A., Schrage B. R., Ziegler C. J., White T. A. (2020). Isolating substituent
effects in Re­(I)-phenanthroline electrocatalysts for CO2 reduction. Inorg. Chim. Acta.

[42] Clark M. L., Cheung P. L., Lessio M., Carter E. A., Kubiak C. P. (2018). Kinetic
and Mechanistic Effects of Bipyridine (bpy) Substituent, Labile Ligand,
and Brønsted Acid on Electrocatalytic CO2 Reduction by Re­(bpy)
Complexes. ACS Catal..

[43] Lovrić, M. , Square-wave voltammetry. In Electroanalytical methods: guide to experiments, 2010; pp 121–145.

[44] Scholz, F. ; Inzelt, G. ; Stojek, Z. , Seminal publications in electrochemistry and electroanalysis. In Electroanalytical Methods: Guide to Experiments, 2010; pp 339–342.

[45] Smieja J. M., Kubiak C. P. (2010). Re­(bipy-tBu)­(CO)­3Cl-improved catalytic activity for
reduction of carbon dioxide: IR-spectroelectrochemical and mechanistic
studies. Inorg. Chem..

[46] Johnson F. P., George M. W., Hartl F., Turner J. J. (1996). Electrocatalytic
Reduction of CO2 Using the Complexes [Re (bpy)­(CO) 3L] n (n = + 1,
L= P (OEt) 3, CH3CN; n= 0, L= Cl-, Otf-; bpy= 2, 2 ‘-Bipyridine;
Otf-= CF3SO3) as Catalyst Precursors: Infrared Spectroelectrochemical
Investigation. J. Organomet. Chem..

[47] Sullivan B., Bolinger C., Conrad D., Vining W., Meyer T. J. (1985). One-Electrocn
and 2-Electron Pathways in the Electrocatalytic Reduction of CO2 by
fac-Re (2, 2 0-bipyridine)-(CO) 3Cl. J. Chem.
Soc., Chem. Commun..

[48] Sastre S., Casasnovas R., Munoz F., Frau J. (2016). Isodesmic reaction
for accurate theoretical pKa calculations of amino acids and peptides. Phys. Chem. Chem. Phys..

[49] Sampaio R. N., Grills D. C., Polyansky D. E., Szalda D. J., Fujita E. (2020). Unexpected
Roles of Triethanolamine in the Photochemical Reduction of CO(2) to
Formate by Ruthenium Complexes. J. Am. Chem.
Soc..

[50] Matsubara Y., Grills D. C., Kuwahara Y. (2015). Thermodynamic Aspects of Electrocatalytic
CO2 Reduction in Acetonitrile and with an Ionic Liquid as Solvent
or Electrolyte. ACS Catal..

[51] Matsubara Y. (2017). Standard Electrode
Potentials for the Reduction of CO2 to CO in Acetonitrile–Water
Mixtures Determined Using a Generalized Method for Proton-Coupled
Electron-Transfer Reactions. ACS Energy Letters.

[52] Mukherjee J. S., Inke (2020). Manganese and Rhenium
Tricarbonyl Complexes Equipped with Proton Relays in the Electrochemical
CO2 Reduction Reaction. Eur. J. Inorg. Chem..

[53] Cotton F. A., Kraihanzel C. S. (1962). Vibrational Spectra and Bonding in
Metal Carbonyls.
I. Infrared Spectra of Phosphine-substitutedGroupVI Carbonyls in the
CO Stretching Region. J. Am. Chem. Soc..

[54] Stor G. J., Hartl F., Van Outersterp J. W.
M., Stufkens D. J. (1995). Spectroelectrochemical
(IR, W/Vis) Determination of the Reduction Pathways for a Series of
[Re­(CO)­3­(a = diimine)­L’]­O/+ (L’ = Halide, Otf, THF,
MeCN, n-PrCN, PPh3, P­(OMe)­3) Complexes. Organometallics.

[55] Yoke
Foo Lee, Kirchhoff Jon R., Bergerb Robert M., Gosztola D. (1995). Spectroelectrochemistry and excited-state
absorption spectroscopy of rhenium (I) α, α′-diimine
complexes. Dalton Trans..

[56] Howell S. L., Gordon K. C. (2006). Vibrational spectroscopy of reduced Re (I) complexes
of 1, 10-phenanthroline and substituted analogues. J. Phys. Chem. A.

[57] Yukiko
Hayashi, Kita Shouichi, Brunschwig Bruce S., Fujita E. (2003). Involvement-of-a-binuclear-species-with-the-re-c­(o)­o-re-moiety-in-co2-reduction-catalyzed-by-tricarbonyl-rhenium­(i). J. Am. Chem. Soc..

[58] Klein A., Vogler C., Kaim W. (1996). The-δ-in-18-δ-electron-complexes-importance-of-the-metal-ligand-interface-for-the-substitutional-reactivity-of-re­(0). Organometallics.

[59] Kaim W., Kohlmann S. (1987). Epr evidence for related electronic structures of α-diimine
complexes with [Ru (bpy) 2] 2+ and re (CO) 3 (halide) fragments. Chem. Phys. Lett..

[60] Hayashi Y., Kita S., Brunschwig B. S., Fujita E. (2003). Involvement of a Binuclear
Species with the Re-C­(O)­O-Re Moiety in CO2 Reduction Catalyzed by
Tricarbonyl Rhenium­(I) Complexes with Diimine Ligands: Strikingly
Slow Formation of the Re-Re and Re-C­(O)­O-Re Species from Re­(dmb)­(CO)­3S
(dmb) 4,4′-Dimethyl-2,2′-bipyridine, S) Solvent). J. Am. Chem. Soc..

[61] Takeda H., Koike K., Inoue H., Ishitani O. (2008). Development of an efficient
photocatalytic system for CO2 reduction using rhenium (I) complexes
based on mechanistic studies. J. Am. Chem. Soc..

[62] Frank P., MichaeláHodges (1993). Excited-state
properties and reactivity of [ReCL (CO) 3 (2, 2′-bipy)]­(2,
2′-bipy= 2, 2′-bipyridyl) studied by time-resolved infrared
spectroscopy. Dalton Trans..

[63] Fujita E., Muckerman J. T. (2004). Why Is Re–Re Bond Formation Cleavage in [Re­(bpy)­(CO)­3]­2
Different. Inorg. Chem..

[64] Benson E. E., Kubiak C. P. (2012). Structural investigations
into the deactivation pathway
of the CO2 reduction electrocatalyst Re­(bpy)­(CO)­3Cl. Chem. Commun..

[65] Mestroni G., Camus A., Mestroni E. (1970). Cobalt complexes
of 2, 2′-bipyridine
and 1, 10-phenanthroline: I. Reaction with alkyl halides and π-acids. J. Org. Chem..

[66] Keene F. R., Creutz C., Sutin N. (1985). Reduction of carbon
dioxide by tris
(2, 2′-bipyridine) cobalt (I). Coord.
Chem. Rev..

[67] Zhang Z., Li Q.-s., Xie Y., King R. B., Schaefer H. F. (2010). Binuclear
and Trinuclear Chromium Carbonyls with Linear Bridging Carbonyl Groups:
Isocarbonyl versus Carbonyl Bonding of Carbon Monoxide Ligands. J. Phys. Chem. A.

