# Supplementary material for: Mechanistic Insights into CO2‑to-CO Photoreduction by Proton-Responsive Imidazole–Pyridine Re(I) Complexes
Source: Inorg Chem. 2026 Apr 16;65(16):9188–98. doi: 10.1021/acs.inorgchem.6c00967 (PMC13126634; doi:10.1021/acs.inorgchem.6c00967)
Supplement: Supplementary file 1 [file ic6c00967_si_001.pdf]

Supporting Information

Mechanistic Insights into CO_2-to-CO Photoreduction by Proton-Responsive Imidazole–Pyridine Re(I) Complexes

Marcos E. G. Carmo[a], Gabrielly Moreira[a], Vanessa F. Silva [a] Jaqueline C. Desordi[b,c], Pablo J. Gonçalves[b,c], Antonio E. Hora Machado[a,d] Renato N. Sampaio[e], Gerald J. Meyer[e] and Antonio O. T. Patrocinio*[a, b]

[a] Laboratory of Photochemistry and Materials Science, Institute of Chemistry, Federal University of Uberlândia, Uberlândia, MG, Brazil. 38400-902. *E-mail:otaviopatrocinio@ufu.br

[b] Centro de Excelência em Hidrogênio e Tecnologias Energéticas Sustentáveis (CEHTES), Goiânia, GO, Brazil. 74690-631.

[c] Instituto de Física, Universidade Federal de Goiás, Goiânia, Brazil. 74690-900.

[d] Programa de Doutorado em Ciências Exatas e Tecnológicas, Universidade Federal de Catalão – UFCat, Catalão 75704-020, Goiás, Brazil.

[e] Department of Chemistry, University of North Carolina at Chapel Hill, Chapel Hill, NC 27599 USA.

Table of Content

S1. Synthesis and characterization methods....2
S2. BIH oxidation scheme and additional structural characterization 6
S3. TD-DFT calculations 7
S4. Time-resolved photoluminescence measurements....9
S5. Lovric's study 10
S6. Complementary photocatalytic measurements 12
S8. Complementary SEC and *in-situ* measurements....14
S9. Data for computed stationary points 18
S10. References....27

S1. Synthesis and characterization methods

Materials. All solvents were HPLC grade. For electrochemical measurements, CH_3CN was dried prior to use (water content <0.001%). The $Re(CO)_5Cl$, 2-(2-pyridy)benzimidazole (pbiH), 2,6-bis(2-benzimidazolyl)pyridine (bbzp) and tetrabutylammonium hexafluorophosphate ($TBAPF_6$) were purchased from Sigma-Aldrich and used as received. 1,3-dimethyl-2-phenyl-2,3-dihydro1H-benzo[d]imidazole (BIH) was synthesized as previously reported.[1, 2]

Synthesis of Re-pbiH and Re-bbzp complexes. The Re(I) complexes with general formula *fac*-$Re(CO)_3(NN)Cl$, was carried following the procedure reported for other Re(I) complexes.[3-6] [$Re(CO)_5Cl$] (0.27 mmol)[6] and the pbiH or bbzp ligand (0.33 mmol) were refluxed in toluene for 3 hours. The mixture was hot-filtered, washed with ethyl ether and the solid was dried under vacuum. For **Re-pbiH**: Yield: 94%. ^1H NMR (($(CD_3)_2SO$) 400 MHz, δ/ppm): 9.08 (d, J = 5.4 Hz, 1H), 8.54 (d, J = 7.9 Hz, 1H), 8.42 (t, J = 7.8 Hz, 1H), 7.88-7.75 (m, 3H), 7.62-7.53 (m, 2H). (–)–HRESIMS *m/z* 499.9803 [M–H]$^-$ (calculated for $C_{15}H_8ClN_3O_3Re^-$, 499.9817 (Δ 2.80 ppm)). Anal. Calcd. for $C_{15}H_9ClN_3O_3Re$ C, 35.97%; H, 1.81%; N, 7.08%. Found: C, 36.10%; H, 1.73%; N, 7.13%. For **Re-bbzp**: Yield: 80.8%. ^1H NMR ($(CD_3)_2SO$) 400 MHz, δ/ppm): 8.69 (d, J = 7.9 Hz, 1H), 8.57 (t, J = 7.9 Hz, 1H), 8.10 (d, J = 7.7 Hz, 1H), 7.93-7.85 (m, 2H), 7.82 – 7.53 (m, 4H), 7.32 (d, J = 5.8 Hz, 2H. (–)–HRESIMS *m/z* 616.0172 [M–H]$^-$ (calculated for $C_{22}H_{12}ClN_5O_3Re^-$, 616.0191 (Δ –3.08 ppm)). Anal. Calcd. for $C_{22}H_{13}ClN_5O_3Re$ C, 42.82%; H, 2.12%; N, 11.35%. Found: C, 42.63%; H, 2.38%; N, 11.72%.

Synthesis of BIH and BID. The (1,3-dimethyl-2-phenyl-2,3dihydro-1H-benzo[d]imidazole) and its deuterated structure were prepared as previously reported.[7, 8] First, 2-phenyl-1,3-dimethylbenzimidazolium iodide was obtained from a two-step methylation reaction of 2-phenylbenzimidazole. The solid was reduced in the presence of

$NaBH_4$ or $NaBD_4$ to yield BIH or BID, respectively. **BIH**: ^{1}H NMR (400 MHz, DMSO-d^6, δ): 7.55 (dd, J = 6.4, 1.9 Hz, 2H), 7.45 (dd, J = 5, 19 Hz, 3H), 6.62 (dd, J = 5.4, 3.3 Hz, 2H), 6.45 (dd, J = 5.4, 3.2 Hz, 2H), 4.87 (s, 1H), 2.48 (s, 6H). **BID**: ^{1}H NMR ^{1}H NMR (400 MHz, DMSO-d^6, δ): 7.55 (dd, J = 6.4, 1.9 Hz, 2H), 7.45 (dd, J = 5, 19 Hz, 3H), 6.62 (dd, J = 5.4, 3.3 Hz, 2H), 6.45 (dd, J = 5.4, 3.2 Hz, 2H), 2.48 (s, 6H).

Structural Characterization. Attenuated total reflectance Fourier-transformed infrared (ATR-FTIR) spectra were recorded in a Perkin Elmer Frontier spectrometer equipped with a diamond crystal plate, using 16 scans at a resolution of 2 cm^{-1}. ^{1}H NMR spectra were recorded in a Bruker Ascend 400 MHz spectrometer using the residual solvent signal as internal standard. High-resolution mass spectra (HRESIMS) with electrospray ionization were measured on an Agilent QTOF (6520B model) spectrometer, operating in the negative mode. Elemental analysis was carried out in a Perkin Elmer 2400 CHNS analyzer.

Photophysical Characterization. Electronic absorption spectra were recorded in a Thermo Evolution 201 spectrophotometer. Room temperature emission measurements were performed in argon degassed CH_3CN solutions in a 1.000 cm quartz cuvette using a Horiba Fluorolog 3 fluorimeter equipped with a standard tungsten-halogen lamp. Emission quantum yields were determined taking *fac*-[Re(CO)$_3$(bpy)Cl], as standard (ϕ_{em} = 0.006 in CH_3CN at 298 K).[9] For emission measurements at 77 K the samples were dissolved in degassed 5:4 (v/v) butyronitrile. Nanosecond time-resolved photoluminescence decays were collected using a Photon Technology International (PTI) GL-301 nitrogen dye laser with a 380 nm excitation. The PL signal was collected using a ScienceTech Model 9010 monochromator and signal enhanced by a Hamamatsu R928 photomultiplier tube and digitalized using a LeCroy Waverunner 4024HD oscilloscope. The singlet oxygen quantum yield (Φ_Δ) was determined by monitoring the singlet oxygen

phosphorescence emission spectrum in a custom-designed system equipped with a cooled InGaAs detector (DSS-IGA 020L - Horiba) coupled to a spectrometer (iHR320 - Horiba). The third harmonic (λ_{exc} = 355 nm) from a Nd:YAG Brilliant Quantel System (Q-switched, 10 Hz repetition rate, ~7 ns FWHM) was used as excitation source. The Φ_Δ values were calculated relative to the *fac*-[Re(CO)$_3$(bpy)Cl, (Φ_Δ = 0.28) standard,[4] using the **Equation S1**. I and I_0 represent the integrals of the intensities of the singlet oxygen emission spectra for the studied samples and the standard, respectively. A and A_0 denote the respective absorbances of the sample and the standard solutions at the excitation wavelength (355 nm). The absorbance values of the samples at the excitation wavelength were adjusted to approximately 0.2. All experiments were conducted at room temperature (25 ± 1°C) and under O_2-saturated conditions.

$$\Phi_\Delta = \left(\frac{I}{I_0}\right)\left(\frac{1-10^{-A_0}}{1-10^{-A}}\right)\Phi_{\Delta_0} \qquad \textbf{(S1)}$$

Electrochemical Measurements. Electrochemical measurements were obtained in a µAutolab PGSTAT204 potentiostat/galvanostat (Autolab) using a glassy carbon as working electrode, a platinum wire as counter electrode, and a silver wire as pseud-reference electrode. All measurements were carried out in Ar or CO_2 saturated anhydrous CH_3CN with tetrabutylammonium hexafluorophosphate (0.1 mol L^{-1}) as a supporting electrolyte. Potentials are reported *vs.* Fc^+/Fc (E^0 = 0.400 V *vs*. SHE). Cyclic voltammetry measurements were carried out with a scan rate of 100 mV s^{-1}. Square Wave Voltammetry (SWV) measurements were performed on Re-bbzp and *fac*-Re(CO)$_3$(bpy)Cl complexes over a frequency range of 50-400 Hz to determine the number of electrons involved in each reduction process. The Lovric method (**Equation S2**) was employed for these calculations, where the αn value was obtained from the linear plot of peak potential (Ep) *versus* log(*f*). The α value was fixed at 1.0, based on the well-characterized *fac*-

[Re(CO)$_3$(bpy)Cl], bpy = 2,2'-bipyridine, complex, for which the number of electrons involved in each process is well established in the literature.

$$\frac{\Delta E}{\Delta log\, f} = \frac{-2.3RT}{\alpha nF} \quad \textbf{(S2)}$$

Computational Methods. Density Functional Theory (DFT) calculations were performed with the Gaussian 09W software using the m06 functional and jorge-TZP-DKH basis to geometry optimization. The oscillator strength for the main electronic transitions were obtained by the time-dependent Density Functional Theory (TD-DFT) using m06 functional and jorge-TZP-DKH basis for Re-bbzp and def2-TZVP for Re-pbiH. All calculations were performed using an IEFPCM continuum solvation model and considering a solvent with inherent properties of CH_3CN.[10]

For the isodesmic reaction, $BIH^{\bullet +}$ + *fac*-[Re^0(CO)$_3$(bbzp)] ⇌ $BI^{\bullet}$ + *fac*-[Re^0(CO)$_3$(bbzpH^+)], the DFT-computed free energies for each species were calculated considering acetonitrile as solvent (see computed stationary points in supporting information). Based on **Equations S3-S5** and the previously reported pK_a for $BIH^{\bullet +}$ of 14.4, the values for $\Delta G_{(reaction)}$, $\Delta G_{(fac\text{-}[Re0(CO)3(bbzpH+)])}$ and pK_a for *fac*-[Re^0(CO)$_3$(bbzpH^+)] were calculated to be 6.93 kcal mol^{-1}, 25.13 kcal mol^{-1} and 19.9, respectivelly.

$$\Delta G_{(reaction)} = (G_{(fac-[\mathrm{Re0(CO)3(bbzpH+)}])} + G_{(\mathrm{BI\bullet})}) - (G_{(fac-[\mathrm{Re0(CO)3(bbzp)}])} + G_{(\mathrm{BIH\bullet +})}) \quad \textbf{(S3)}$$

$$\Delta G_{(reaction)} = \Delta G_{(fac-[\mathrm{Re0(CO)3(bbzpH+)}])} - \Delta G_{(\mathrm{BIH\bullet +})} \quad \textbf{(S4)}$$

$$\Delta G = 1.264\, pK_a \quad \textbf{(S5)}$$

S2. BIH oxidation scheme and additional structural characterization

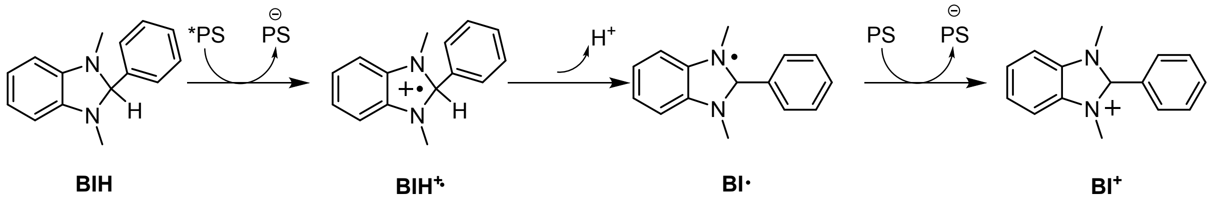

Scheme S1. Oxidation process of the sacrificial donor BIH in the photochemical CO_2 reduction. PS stands for photosensitiser.

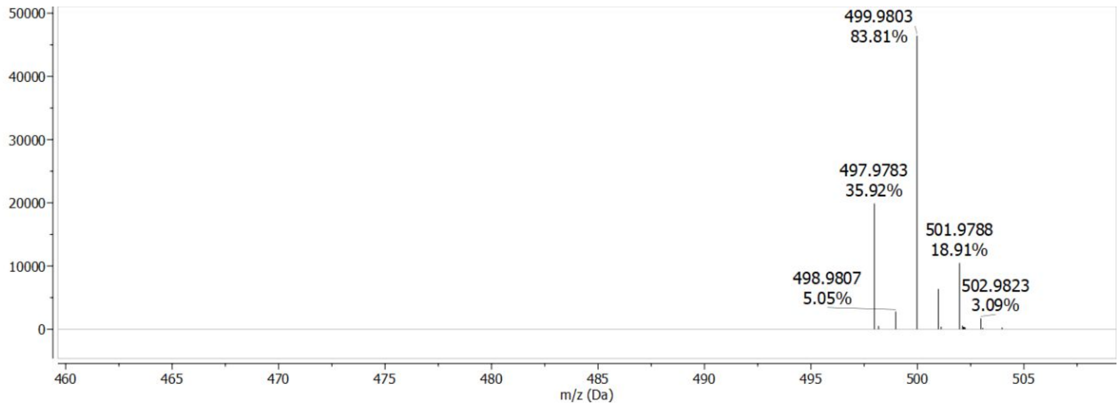

Figure S1. (–)–HRESIMS spectrum of Re-pbiH.

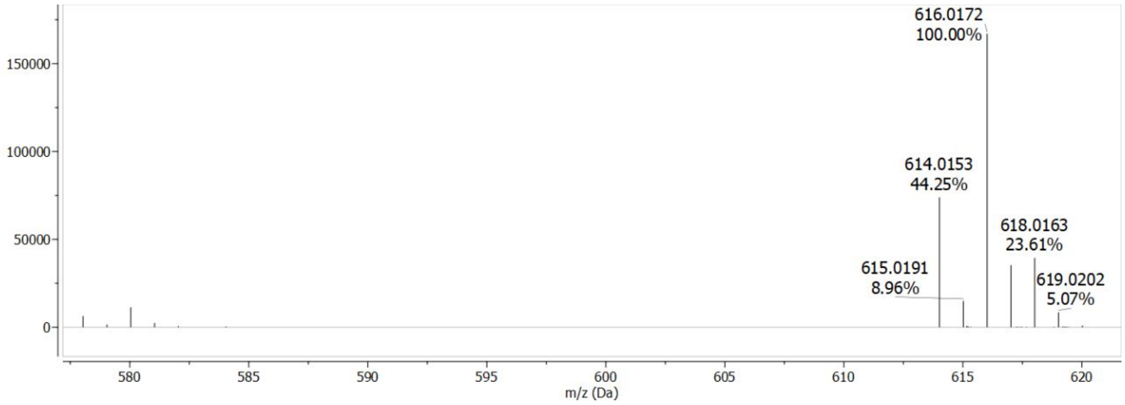

Figure S2. (–)–HRESIMS spectrum of Re-bbzp.

S3. TD-DFT calculations

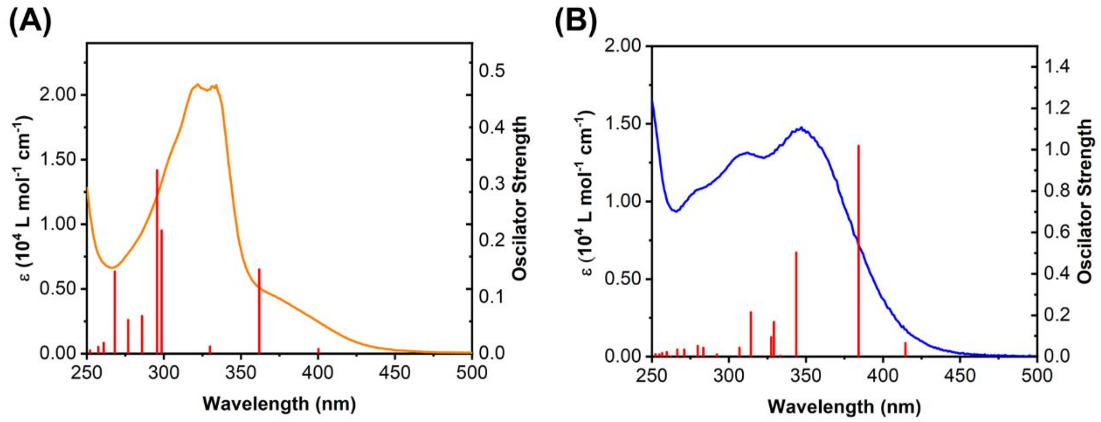

Figure S3. Experimental UV-Vis absorption spectra of Re-pbiH (A) and Re-bbzp (B) complexes compared to the these TD-DFT predicted oscillators strengths (right axis).

Table S1. Lowest energy electronic transitions and their corresponding oscillator strengths for the main UV-Vis transitions found for Re-pbiH.

l nm (eV)	Oscillator strength	Transitions	Nature
400.33 (3.0971)	0.0084	H-1 → L (8.2%)<br>H → L (91.8%)	$MLCT_{Re \to NN}$
361.81 (3.4268)	0.1494	H-1 → L (92.2%)<br>H → L (7.8%)	$MLCT_{Re \to NN}$
329.82 (3.7592)	0.0127	H-2 → L (92.1%)<br>H-2 → L+1 (2.8%)<br>H-2 → L+2 (2.8%)<br>H-2 → L+3 (2.2%)	$MLCT_{Re \to NN}$

Table S2. Lowest energy electronic transitions and their corresponding oscillator strengths for the main UV-Vis transitions found for Re-bbzp.

l nm (eV)	Oscillator strength	Transitions	Nature
359.28 (3.4510)	0.0345	H-1 → L (51.6%)<br>H → L (48.4%)	$MLCT_{Re \to NN}$

343.44 (3.6101)	0.8567	H-4 → L (4.3%)<br>H-1 → L (41.7%)<br>H → L (54.0%)	$MLCT_{Re\rightarrow NN}$
305.40 (4.0598)	0.6716	H-4 → L+1 (2.6%)<br>H-3 → L (66.1%)<br>H-2 → L (20.5%)<br>H-1 → L (3.7%)<br>H-1 → L+1 (7.0%)	$MLCT_{Re\rightarrow NN}$

S4. Time-resolved photoluminescence measurements

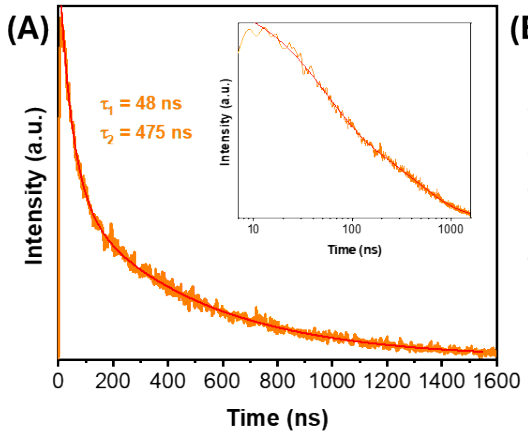

Figure S4. Emission lifetimes measurements for Re-pbiH (a) and Re-bbzp (b) in in CH_3CN at 298 K. *Inset:* log(t) vs I plot of the decays.

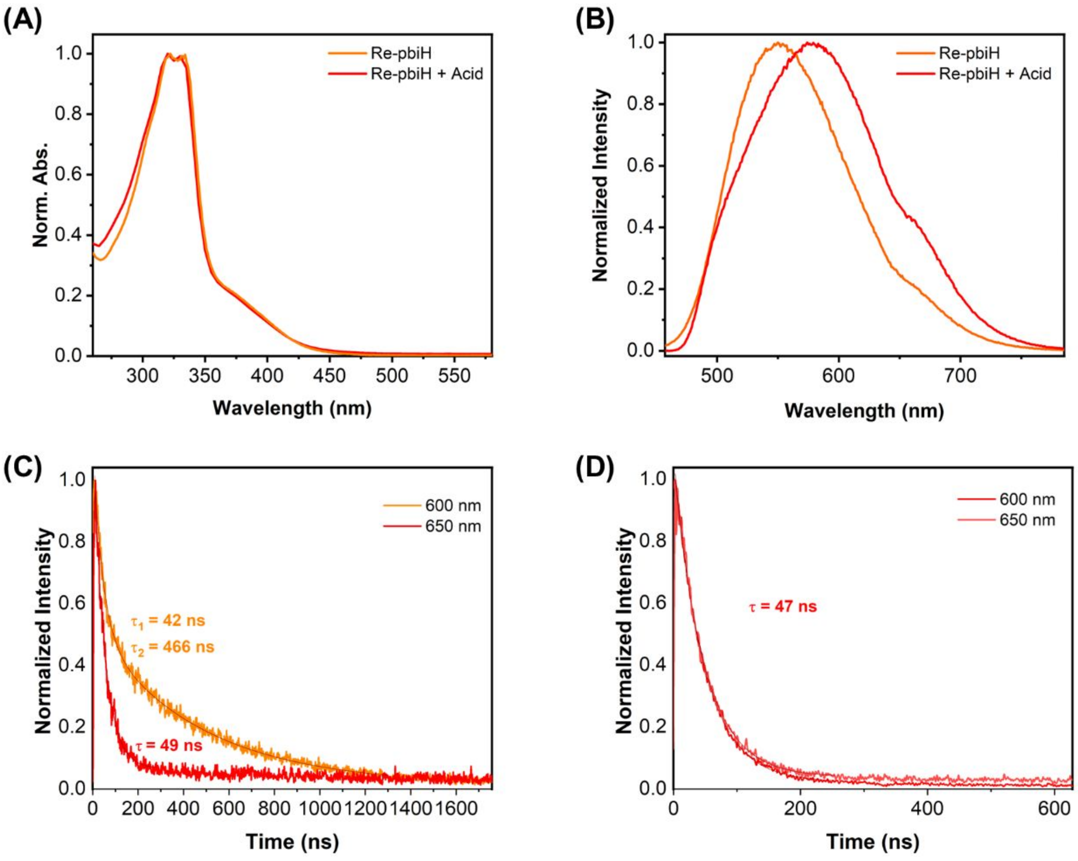

Figure S5. Absorption spectra (A) and emission spectra (B) of pure Re-pbiH in CH_3CN and after addition of $HClO_4$ (acid). Emission decay (C) for Re-pbiH emission monitored at different wavelengths (C) and emission decay for Re-pbiH in CH_3CN and after addition of $HClO_4$ (acid) (D).

S5. Lovric's study

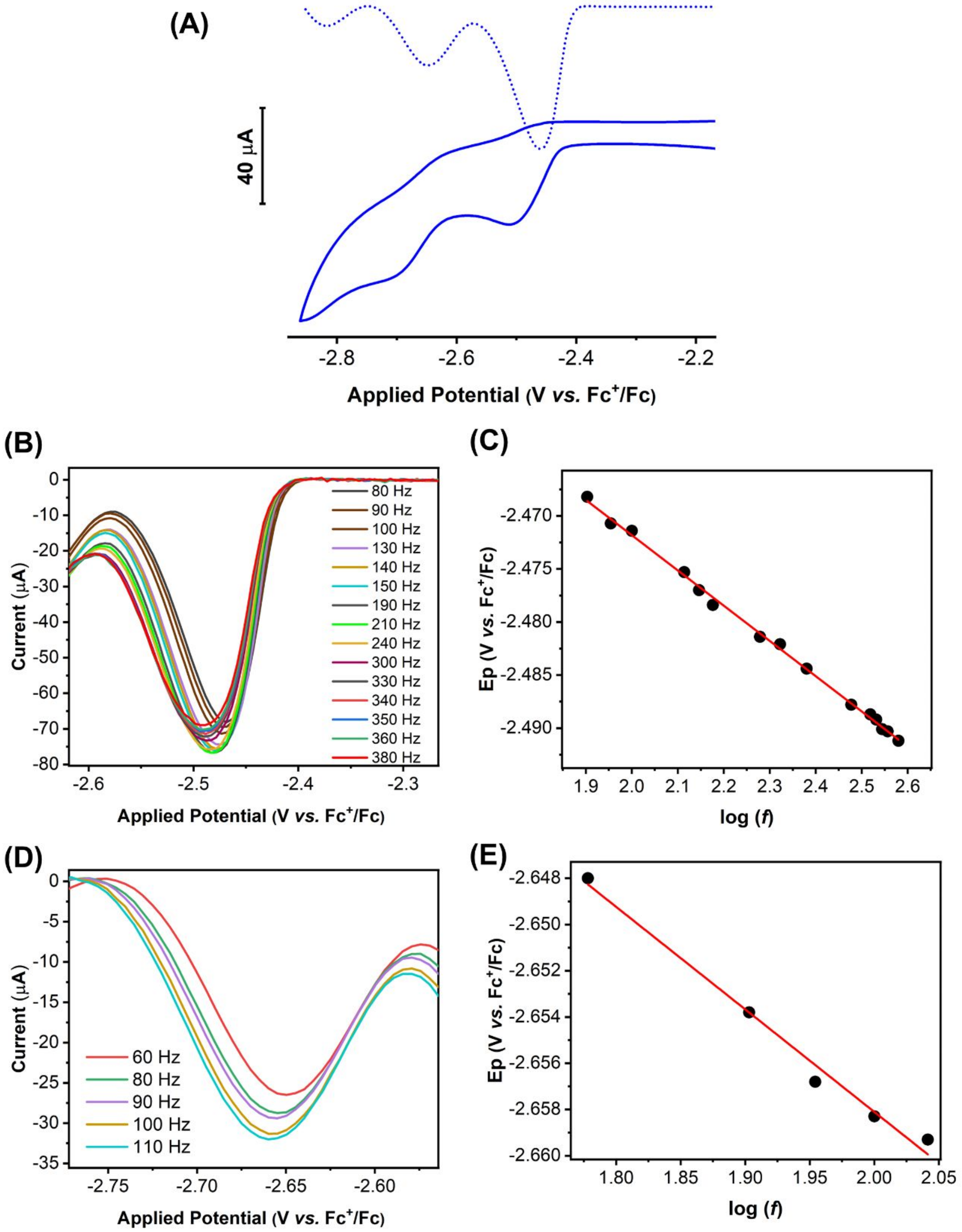

Figure S6. (a) Cyclic Voltammogram at 100 mV s^{-1} of scan rate (solid line) and Square Wave Voltammetry at 50 Hz of frequency (dashed line) for Re-bbzp complex. (b) and (c) are showing the SWV as a function of applied frequency (*f*) variation as well as its linear dependence along to the applied frequency for the first reduction process (n = 2.0),

respectively. (d) and (e) are showing the SWV as a function of applied frequency (*f*) variation as well as its linear dependence along to the applied frequency for the second reduction process (n = 1), respectively.

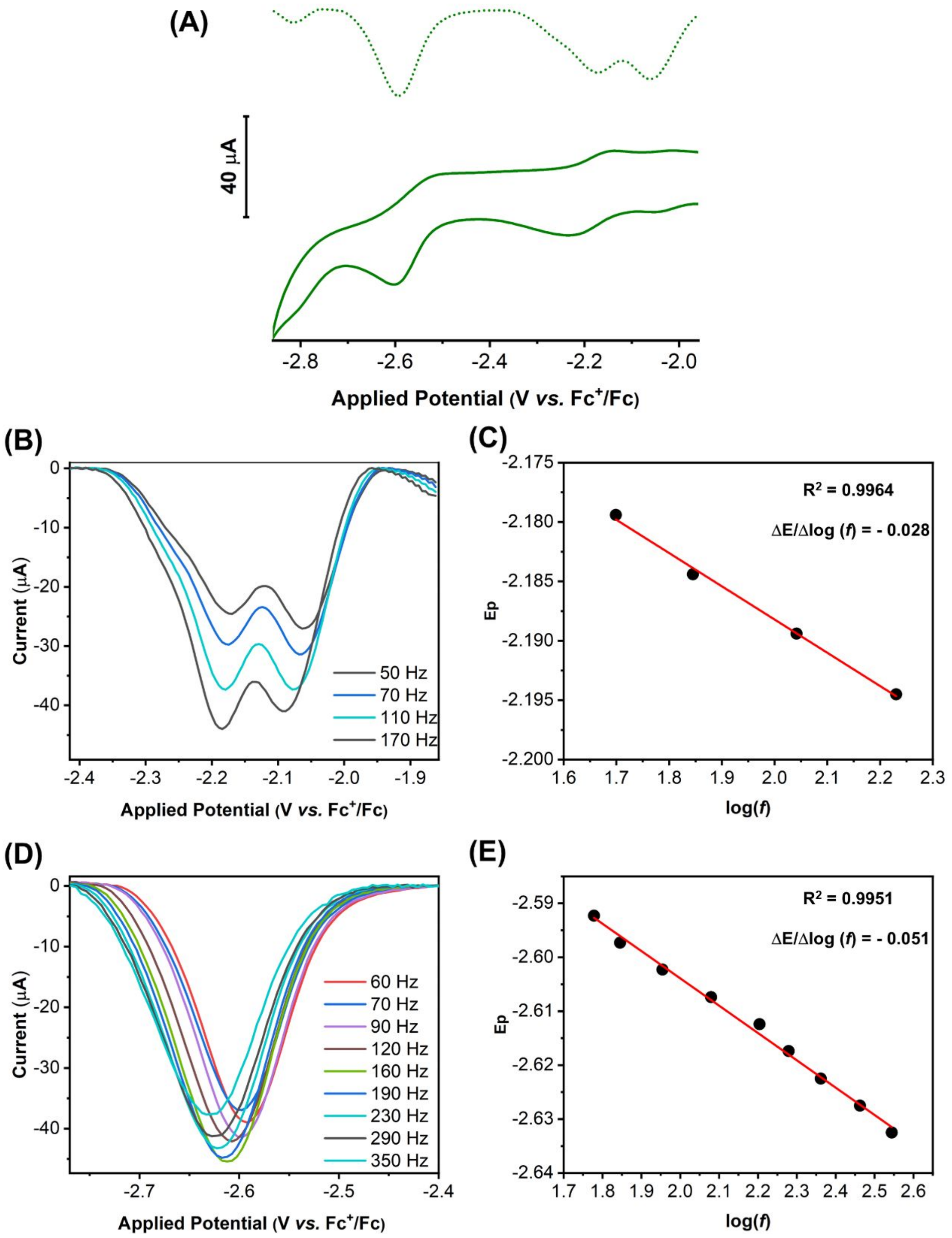

Figure S7. (a) Cyclic Voltammogram at 100 mV s^{-1} of scan rate (solid line) and Square Wave Voltammetry at 50 Hz of frequency (dashed line) for *fac*-$Re(CO)_3$(bpy)Cl complex.

(b) and (c) are showing the SWV as a function of applied frequency (*f*) variation as well as its linear dependence along to the applied frequency for the first reduction process (n = 2.0), respectively. (d) and (e) are showing the SWV as a function of applied frequency (*f*) variation as well as its linear dependence along to the applied frequency for the second reduction process (n = 1.0), respectively.

S6. Complementary photocatalytic measurements

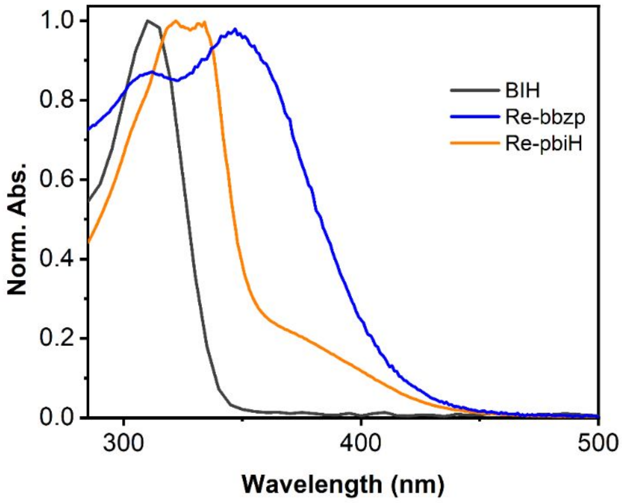

Figure S8. UV-vis spectra of the used electron donor (BIH) and evaluated photocatalysts in acetonitrile at 298 K.

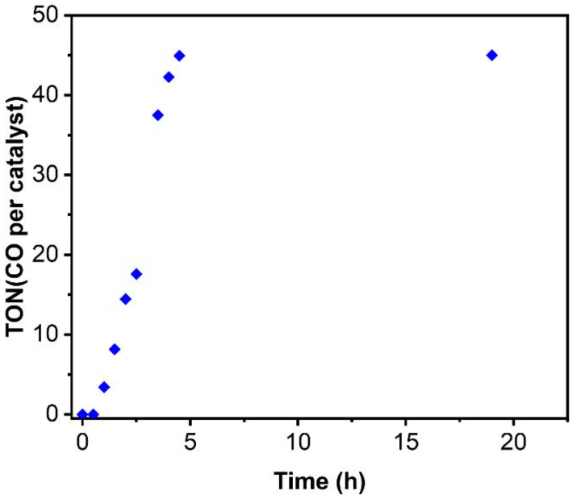

Figure S9. Long-term irradiation time dependence of CO formation photocatalyzed by Re-bbzp complex. Experiments were performed in CO_2-saturated 10 mL CH_3CN

solutions containing 6.7 mmol L^{-1} of BIH and 0.03 mmol L^{-1} of photocatalysts ($\lambda > 370$ nm).

Table S3. Photocatalytic performance of studied complexes in CH_3CN solution containing 0.03 mmol L^{-1} of complex.

Complex	Donor agent	Irradiation time	TONco	TON_{H2}	CO Selectivity
**$Re(CO)_3$(pbiH)Cl**	BIH/TEOA[a]	3 h	4	1	75 %
**$Re(CO)_3$(pbiH)Cl**	BIH[b]	3 h	8	0	100 %
**$Re(CO)_3$(bbpz)Cl**	BIH/TEOA[a]	3 h	0	0	-
**$Re(CO)_3$(bbpz)Cl**	BIH[b]	3 h	30	0	100 %

[a] CH_3CN/TEOA 5:1 v/v. [b] 6.7 mmol L^{-1} of BIH.

S7. Complementary details for ligand-assisted proton transfer study

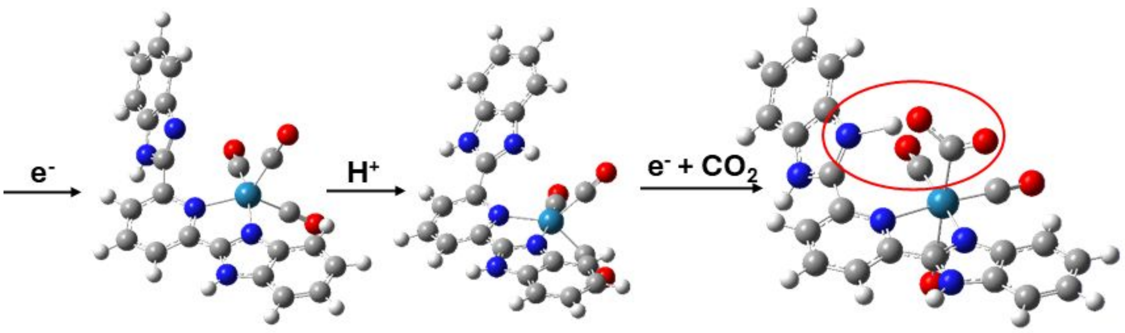

Figure S10. DFT-optimized geometry of the two-electron- and one-proton-reduced Re-bbzp species featuring a CO_2 adduct intermediate.

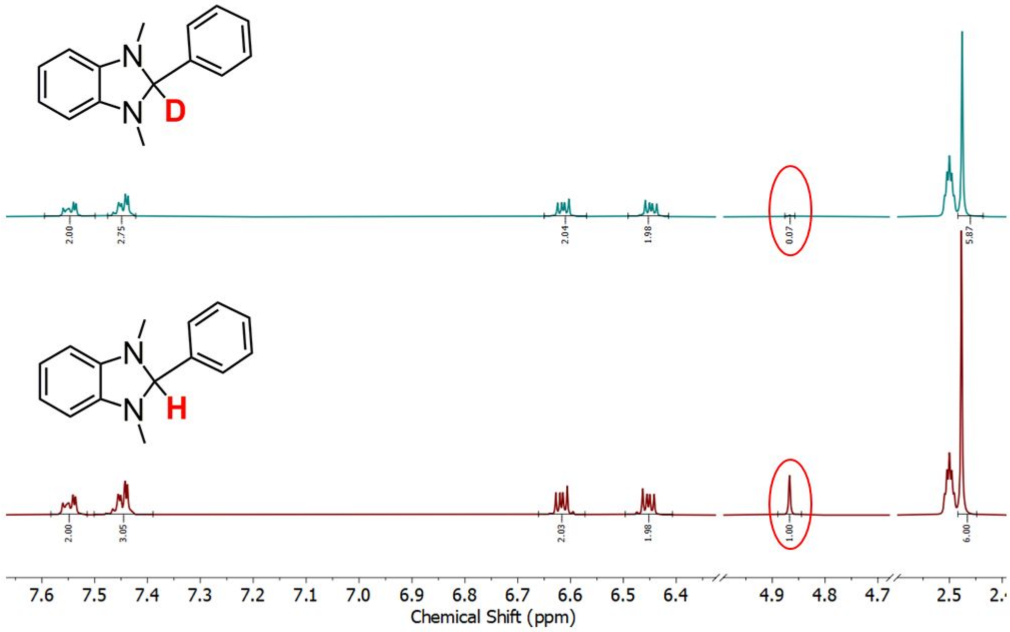

Figure S11. ^{1}H-NMR spectra of the complex BIH (red) and its deuteride form (BID) (blue) in DMSO-d[6]. The highlighted peak corresponds to the replaced proton on BIH.

S8. Complementary SEC and *in-situ* measurements

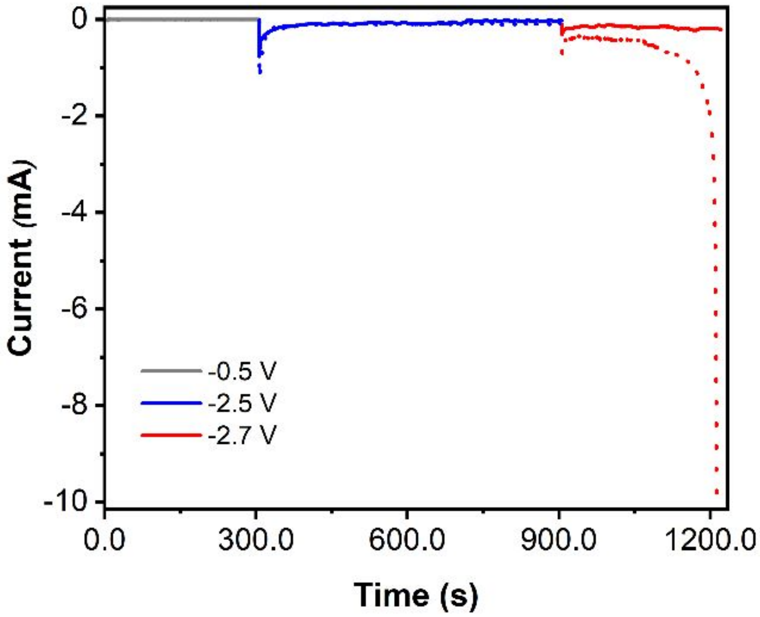

Figure S12. Chronoamperometry measurements of Re-bbzp complex performed for the IR-SEC experiments in acetonitrile with Bu_4NPF_6 electrolyte for the first (E□ = –2.5 V

vs. Fc^+/Fc) and second (E□ = –2.7 V vs. Fc^+/Fc) electron reduction process under Ar (solid line) and CO_2 (dotted line) atmosphere. The potential -0.5 V *vs*. Fc^+/Fc did not show changes in the IR spectra as expected.

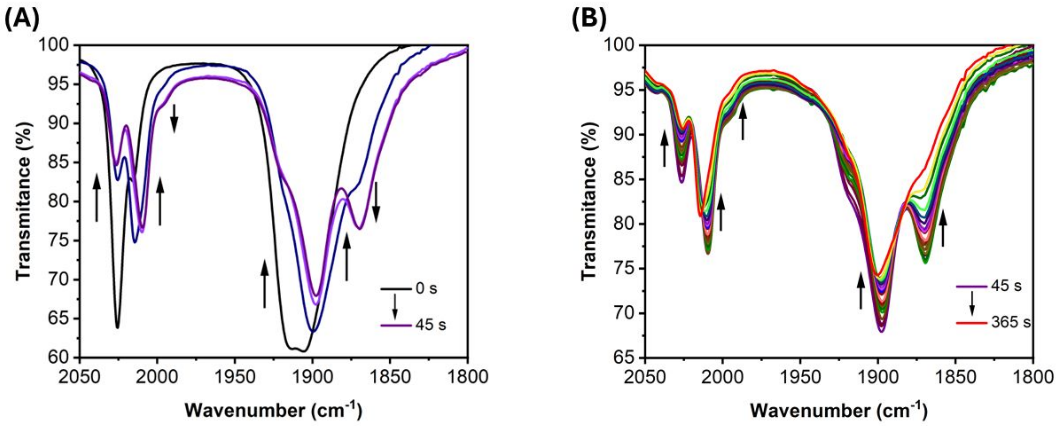

Figure S13. IR-SEC of Re-bbzp in acetonitrile with Bu_4NPF_6 electrolyte for the first electron reduction process under Ar atmosphere (Δt = 15s).

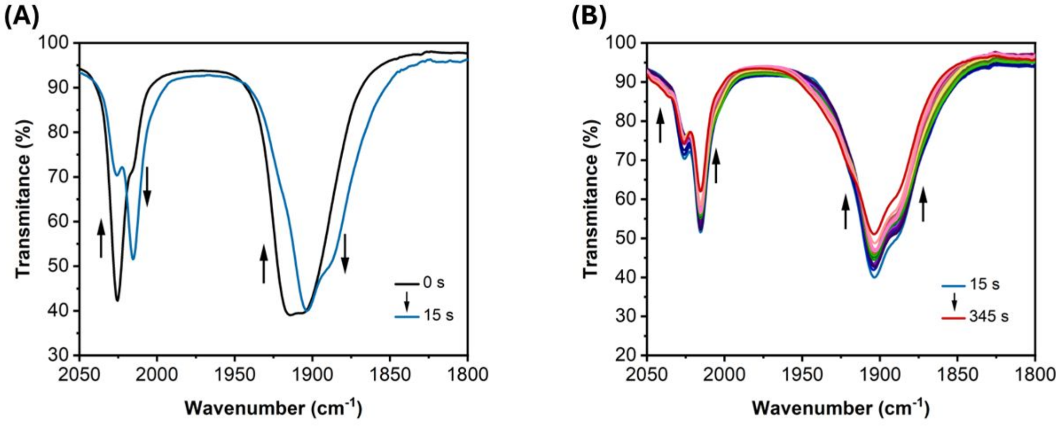

Figure S14. IR-SEC of Re-bbzp in acetonitrile with Bu_4NPF_6 electrolyte for the first electron reduction process under CO_2 atmosphere (a) t < 15 s (b) t > 15 s. (Δt = 15s).

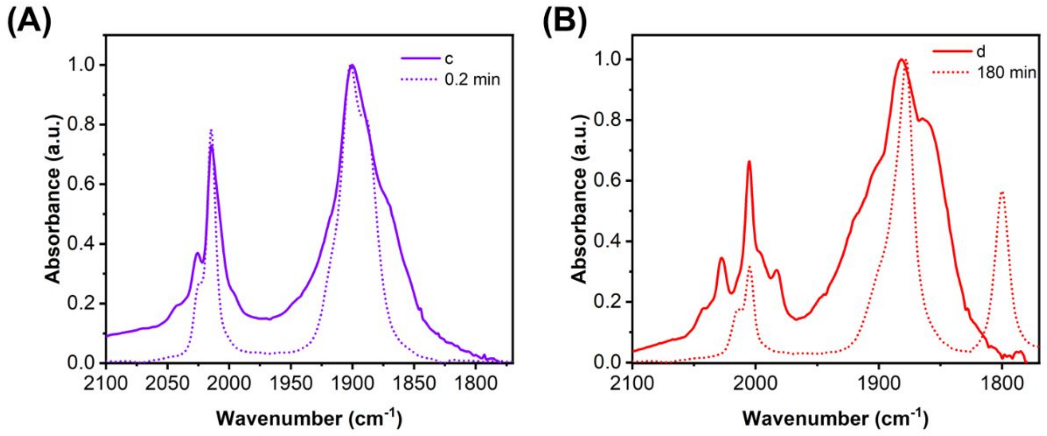

Figure S15. Comparison between the *in-situ* experimental spectra (dotted lines) and the corresponding spectroelectrochemical (SEC) spectra (solid lines) of one-(a) and two- (b) electron reduced species under Ar atmosphere.

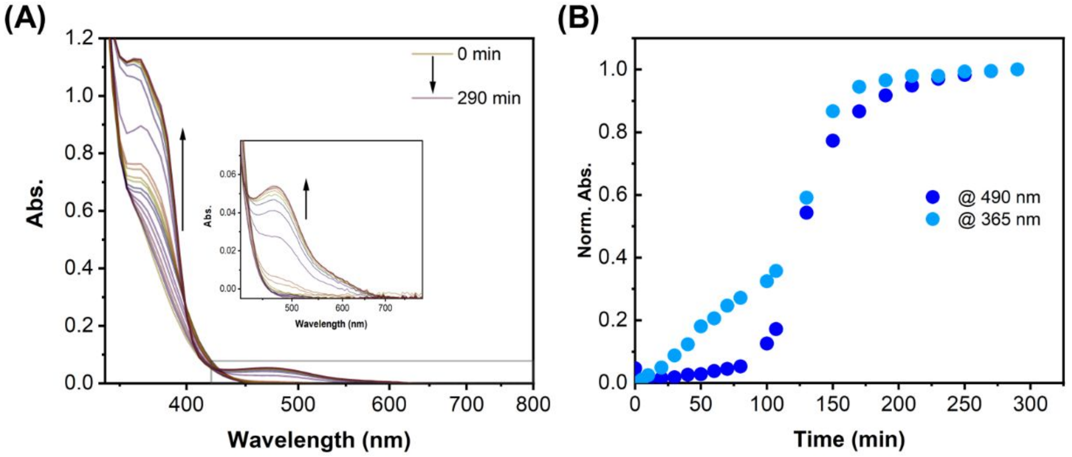

Figure S16. UV−vis spectral changes (a) and the corresponding kinetic traces at selected wavelengths during the photoirradiation for Re-bbzp (0.06 mmol L^{-1}) in Ar-saturated CH_3CN solutions containing excess of BIH (1.40 mmol L^{-1}) (λ_{irr} = 365 nm).

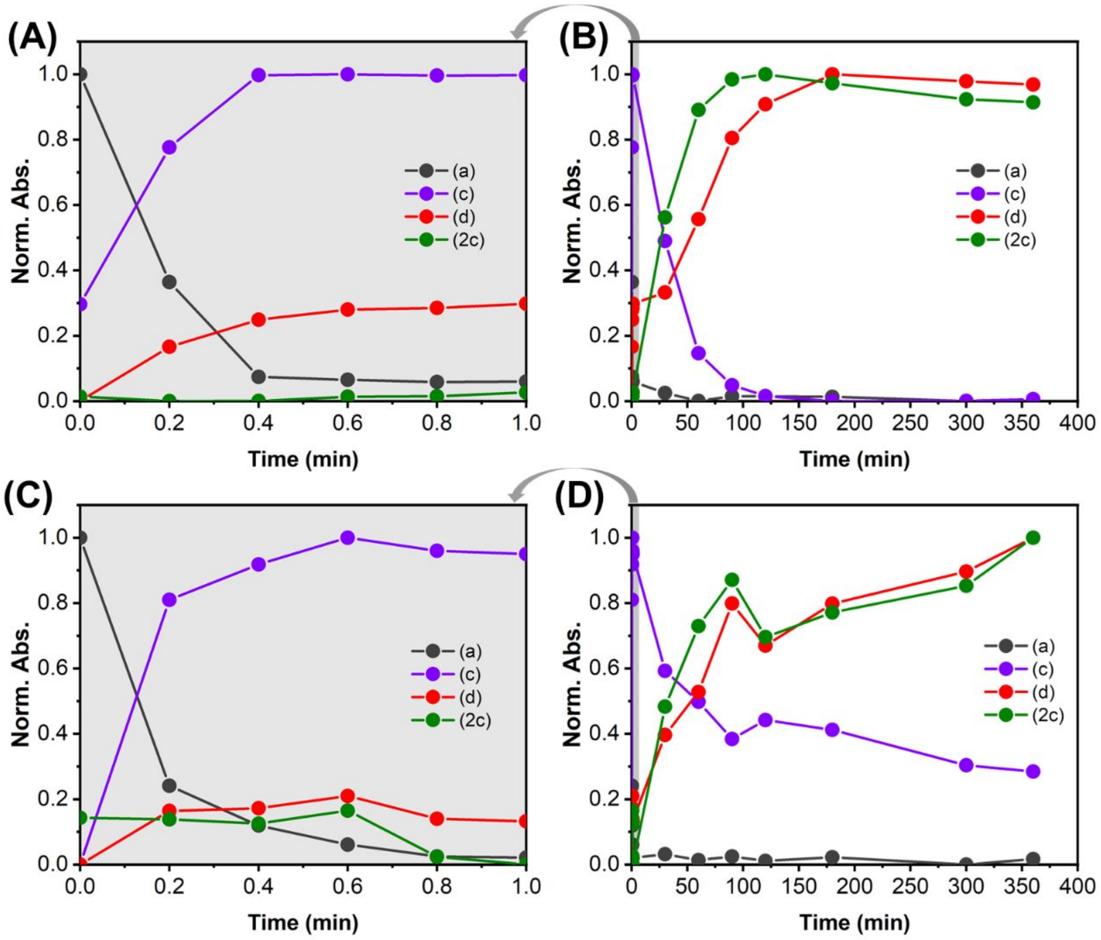

Figure S17. kinetic monitoring for each species based their corresponding peaks (2025 (a), 2015 (c), 2005 (d) and 1800 cm^{-1} for (2c)) under Ar (A and B) and CO_2 (B and C) atmosphere.

S9. Data for computed stationary points

fac-[Re(CO)$_3$(bbzp)]

Charge: 0 Multiplicity: 2

C	1.32258600	1.99456800	-0.10124300
C	-1.03455700	2.27199000	-0.00792600
C	1.48297100	3.37154900	-0.19771400
C	-0.89574300	3.67006100	-0.08992400
C	0.36132700	4.23038400	-0.22220600
H	2.48343600	3.78582400	-0.19791800
H	-1.77846700	4.29832500	-0.05759900
C	-2.27535600	1.60130900	0.11982500
H	-3.78656500	3.08191800	0.38347600
N	-2.31208600	0.23647300	0.06876900
Re	-0.46747700	-0.70532200	-0.37012300
C	-1.27138000	-2.33733500	-0.97634900
C	1.17311800	-1.20168500	-1.24935200
C	0.09328000	-1.54787200	1.21243100
O	2.16254700	-1.50474900	-1.83291300
O	-1.78881300	-3.33473500	-1.37019200
O	0.42680500	-2.07606800	2.22637900
N	0.06776300	1.39753500	-0.09338200
N	-3.54432600	2.10758000	0.29935900
C	-4.43520000	1.03756500	0.39441400
C	-3.65809300	-0.14186900	0.25588900

C	-4.26399200	-1.39977200	0.33066600
C	-5.81692100	0.99652800	0.59294700
C	-5.64733300	-1.44569500	0.53095600
C	-6.41204500	-0.26716800	0.65757200
H	-3.68489900	-2.30919700	0.23867200
H	-6.40243300	1.90260600	0.69599000
H	-7.48298700	-0.34134700	0.81173600
H	-6.14211500	-2.40873400	0.59325000
C	4.66581200	0.54723900	-0.18918600
H	3.73898300	1.97512900	-1.49863400
C	4.05982800	-0.13902800	0.89892600
C	2.52740300	1.17429800	0.07651400
N	3.67083700	1.38037200	-0.68686600
N	2.72918300	0.27567600	1.04155500
C	4.79807800	-1.06761900	1.64799300
C	5.99706600	0.32628300	-0.56056500
H	6.45367200	0.85021500	-1.39260600
H	4.34125800	-1.59040500	2.48091800
C	6.12584500	-1.28884000	1.28470200
H	6.72326800	-2.00052900	1.84472700
C	6.71529500	-0.60223000	0.19545600
H	7.75151500	-0.80205300	-0.05645600
H	0.49026000	5.30235900	-0.30150900

Zero-point correction= 0.315407 (Hartree/Particle)

Thermal correction to Energy= 0.340083

Thermal correction to Enthalpy= 0.341027

Thermal correction to Gibbs Free Energy= 0.258606

Sum of electronic and zero-point Energies= -1424.172824

Sum of electronic and thermal Energies= -1424.148148

Sum of electronic and thermal Enthalpies= -1424.147204

Sum of electronic and thermal Free Energies= -1424.229625

***fac*-[Re(CO)$_3$(bbzpH)]$^+$**

Charge: 1 Multiplicity: 2

C	-1.40023900	1.92570500	0.23177300
C	0.94648300	2.22540300	-0.01973100
C	-1.54312000	3.28184500	0.53928900
C	0.82187000	3.58590100	0.24670000
C	-0.42463000	4.12403900	0.58424700
H	-2.53476300	3.70143600	0.65856600
H	1.69526800	4.22618800	0.20360500
C	2.18034200	1.57540200	-0.32956300
H	3.61600500	3.09479700	-0.71631000
N	2.24105000	0.21926300	-0.33156000
Re	0.45326100	-0.75127400	0.34979300
C	0.93681000	-0.75161500	2.16062400
C	-1.26371100	-1.48090200	0.85199200

C	1.11122800	-2.55413500	0.16982800
O	-2.30123600	-1.94899800	1.18236400
O	1.26437700	-0.70608900	3.30210100
O	1.51099900	-3.66438000	0.05316500
N	-0.14068200	1.32948000	0.07461200
N	3.40597400	2.11310800	-0.62449700
C	4.30658900	1.06640400	-0.82999700
C	3.56866400	-0.13150700	-0.64253400
C	4.19503800	-1.37569000	-0.78064100
C	5.66445600	1.06187700	-1.15809000
C	5.55316800	-1.38439500	-1.10583800
C	6.27691400	-0.18597300	-1.29228700
H	3.65163700	-2.30094100	-0.64372800
H	6.21745800	1.98273000	-1.30060100
H	7.33023600	-0.23543100	-1.54479100
H	6.06419000	-2.33404600	-1.21901700
C	-4.75320100	0.48997100	-0.12545500
H	-3.97598100	1.84885900	1.34569200
C	-4.06402800	-0.17195100	-1.16467900
C	-2.58802200	1.16558900	-0.08030500
N	-3.80606200	1.29827700	0.51652200
C	-4.70954500	-1.06924300	-2.01777000
C	-6.11347400	0.27811700	0.10464700
H	-6.64157000	0.78512400	0.90253800

H	-4.18220600	-1.57524600	-2.81659900
C	-6.07169100	-1.28001900	-1.79001100
H	-6.61360700	-1.96732500	-2.42923100
C	-6.76022100	-0.62018600	-0.74804700
H	-7.81701800	-0.81466400	-0.60676800
H	-0.53377100	5.17609400	0.81054800
N	-2.73982200	0.27379600	-1.10028100
H	-2.00484700	0.02840200	-1.74789100

Zero-point correction= 0.329355 (Hartree/Particle)

Thermal correction to Energy= 0.354110

Thermal correction to Enthalpy= 0.355055

Thermal correction to Gibbs Free Energy= 0.272549

Sum of electronic and zero-point Energies= -1424.617030

Sum of electronic and thermal Energies= -1424.592274

Sum of electronic and thermal Enthalpies= -1424.591330

Sum of electronic and thermal Free Energies= -1424.673836

$BIH^{\bullet+}$

Charge: 1 Multiplicity: 2

C	1.70972700	0.00000000	-0.23232400
C	2.87955900	0.00000000	-1.00577300
C	1.79661500	0.00000000	1.17053400
C	4.13324500	0.00000000	-0.38116900

H	2.81305100	0.00000100	-2.08961700
C	3.04855100	0.00000000	1.79028200
H	0.89393500	-0.00000100	1.77286000
C	4.21806500	0.00000000	1.01528800
H	5.03502100	0.00000100	-0.98355800
H	3.11367300	-0.00000100	2.87292600
H	5.18849700	0.00000000	1.50012900
C	0.35800700	0.00000000	-0.92485800
H	0.50796700	0.00000000	-2.01526900
C	-1.72353400	0.72016500	-0.12332400
C	-1.72353400	-0.72016500	-0.12332400
C	-2.86066200	1.44267600	0.28411000
C	-2.86066200	-1.44267600	0.28411000
C	-3.97032300	0.71270000	0.68806800
H	-2.87159400	2.52471700	0.27698700
C	-3.97032300	-0.71270000	0.68806800
H	-2.87159500	-2.52471700	0.27698700
H	-4.86359200	1.23639800	1.00742400
H	-4.86359200	-1.23639800	1.00742300
N	-0.50813900	-1.14842900	-0.55927000
N	-0.50813900	1.14842900	-0.55927000
C	-0.10123700	-2.53274600	-0.79198300
H	0.97977900	-2.61264000	-0.67089100
H	-0.58419400	-3.18402200	-0.06211100

H	-0.37626700	-2.85413400	-1.80241800
C	-0.10123700	2.53274600	-0.79198300
H	0.97977900	2.61263900	-0.67089600
H	-0.37627200	2.85413600	-1.80241600
H	-0.58418900	3.18402100	-0.06210700

Zero-point correction= 0.281170 (Hartree/Particle)

Thermal correction to Energy= 0.295104

Thermal correction to Enthalpy= 0.296048

Thermal correction to Gibbs Free Energy= 0.238953

Sum of electronic and zero-point Energies= -690.117486

Sum of electronic and thermal Energies= -690.103551

Sum of electronic and thermal Enthalpies= -690.102607

Sum of electronic and thermal Free Energies= -690.159703

BI·

Charge: 0 Multiplicity: 2

C	1.75137400	-0.00000300	-0.00000200
C	2.50828100	-1.08247900	-0.56729300
C	2.50827600	1.08247700	0.56728900
C	3.89839600	-1.07385100	-0.55827400
H	1.98959600	-1.90133800	-1.05235600
C	3.89839100	1.07385600	0.55827200
H	1.98958600	1.90133500	1.05235200

C	4.61803700	0.00000400	-0.00000100
H	4.43372000	-1.90571800	-1.00816800
H	4.43371100	1.90572600	1.00816500
H	5.70278100	0.00000700	-0.00000100
C	0.32991600	-0.00000400	-0.00000400
C	-1.85807500	0.70704000	-0.02117400
C	-1.85807700	-0.70704500	0.02116900
C	-3.05031900	1.43037700	-0.04505100
C	-3.05032400	-1.43037700	0.04504600
C	-4.25627000	0.69978000	-0.02446800
H	-3.05693300	2.51389800	-0.06610200
C	-4.25627200	-0.69977400	0.02446600
H	-3.05694400	-2.51389700	0.06609500
H	-5.19886300	1.23697300	-0.04268700
H	-5.19886700	-1.23696400	0.04268600
N	-0.52509200	-1.12617800	0.04720700
N	-0.52509100	1.12617000	-0.04721800
C	-0.13161000	-2.48694600	0.40739300
H	0.80664400	-2.46412200	0.96282300
H	-0.90356000	-2.92259600	1.04757400
H	-0.00666000	-3.12598900	-0.47328200
C	-0.13161400	2.48694800	-0.40736900
H	0.80669400	2.46415000	-0.96270600
H	-0.90351300	2.92257600	-1.04762700

H -0.00676800 3.12599300 0.47332000

Zero-point correction= 0.268111 (Hartree/Particle)

Thermal correction to Energy= 0.282317

Thermal correction to Enthalpy= 0.283261

Thermal correction to Gibbs Free Energy= 0.226658

Sum of electronic and zero-point Energies= -689.662990

Sum of electronic and thermal Energies= -689.648785

Sum of electronic and thermal Enthalpies= -689.647841

Sum of electronic and thermal Free Energies= -689.704444

S10. References

1. Zhu, X.-Q.; Zhang, M.-T.; Yu, A.; Wang, C.-H.; Cheng, J.-P., Hydride, hydrogen atom, proton, and electron transfer driving forces of various five-membered heterocyclic organic hydrides and their reaction intermediates in acetonitrile. *J. Am. Chem. Soc.* **2008,** *130* (8), 2501-2516.
2. Hasegawa, E.; Seida, T.; Chiba, N.; Takahashi, T.; Ikeda, H., Contrastive photoreduction pathways of benzophenones governed by regiospecific deprotonation of imidazoline radical cations and additive effects. *J. Org. Chem.* **2005,** *70* (23), 9632-9635.
3. Souza, B. L.; Faustino, L. A.; Prado, F. S.; Sampaio, R. N.; Maia, P. I. S.; Machado, A. E. H.; Patrocinio, A. O. T., Spectroscopic characterization of a new Re(i) tricarbonyl complex with a thiosemicarbazone derivative: Towards sensing and electrocatalytic applications. *Dalton Trans.* **2020,** *49* (45), 16368-16379.
4. Ramos, L. D.; da Cruz, H. M.; Morelli Frin, K. P., Photophysical properties of rhenium (I) complexes and photosensitized generation of singlet oxygen. *Photochem. Photobiol. Sci.* **2017,** *16*, 459-466.
5. Ramos, L. D.; Sampaio, R. N.; De Assis, F. F.; De Oliveira, K. T.; Homem-De-Mello, P.; Patrocinio, A. O. T.; Frin, K. P. M., Contrasting photophysical properties of rhenium(i) tricarbonyl complexes having carbazole groups attached to the polypyridine ligand. *Dalton Trans.* **2016,** *45* (29), 11688-11698.
6. Sousa, S. F.; Sampaio, R. N.; Barbosa Neto, N. M.; Machado, A. E. H.; Patrocinio, A. O. T., The photophysics of fac-[Re(CO)3(NN)(bpa)]+ complexes: A theoretical/experimental study. *Photochem. Photobiol. Sci.* **2014,** *13* (8), 1213-1224.
7. Sampaio, R. N.; Grills, D. C.; Polyansky, D. E.; Szalda, D. J.; Fujita, E., Unexpected Roles of Triethanolamine in the Photochemical Reduction of CO(2) to Formate by Ruthenium Complexes. *J. Am. Chem. Soc.* **2020,** *142* (5), 2413-2428.
8. Müller, A. V.; Ahmad, S.; Sirlin, J. T.; Ertem, M. Z.; Polyansky, D. E.; Grills, D. C.; Meyer, G. J.; Sampaio, R. N.; Concepcion, J. J., Reduction of CO to Methanol with Recyclable Organic Hydrides. *J. Am. Chem. Soc.* **2024,** *146* (15), 10524-10536.
9. Patrocinio, A. O. T.; Brennaman, M. K.; Meyer, T. J.; Murakami Iha, N. Y., Excited-state dynamics in fac- [Re(CO)3(Me4phen)(L)] +. *J. Phys. Chem. A* **2010,** *114*, 12129-12137.
10. Tomasi, J.; Mennucci, B.; Cammi, R., Quantum mechanical continuum solvation models. *Chem. Rev.* **2005,** *105*, 2999-3093.
